# Mapping bacterial microbiota variations in raw milk: geographic and type-specific insights

**DOI:** 10.1128/spectrum.00933-25

**Published:** 2025-10-27

**Authors:** Yujing Wang, Bowen Li, Rongbo Fan, Qijing Du, Dengpan Bu, Changjiang Zang, Jun Wang, Rongwei Han, Yongxin Yang

**Affiliations:** 1 College of Food Science and Engineering, Qingdao Agricultural University 665564, Qingdao, Shandong, China; 2 College of Animal Science, Xinjiang Agricultural University 665539, Urumqi, Xinjiang, China; University of Turin, Gruglisco, Turin, Italy

**Keywords:** milk microbiota, lactic acid bacteria, Farm disparities, milk types

## Abstract

Milk harbors a diverse array of microorganisms; however, the current understanding of the microbial diversity in raw milk from specific geographical origins and milk types remains unclear. To address this knowledge gap, this study employed single-molecule real-time sequencing to analyze the differences in microbial communities across various farms and milk types from different regions, specifically by examining the microbial diversity in Holstein cow milk samples from farms situated in both adjacent and distant regions, and comparing the microbial diversity between Holstein cow milk and non-bovine milk from farms in adjacent regions. The results demonstrated that several genera, such as *Lactiplantibacillus*, *Pseudomonas*, *Acinetobacter*, and *Chryseobacterium*, exhibited significant differences in Holstein cow milk from adjacent regions. Additionally, genera like *Lactiplantibacillus* and *Chryseobacterium* consistently showed substantial variation in Holstein cow milk across distant regions. At the species level, *Lactiplantibacillus plantarum*, *Pseudomonas lurida*, and *Haloanella gallinarum* displayed significant variations in Holstein cow milk from adjacent regions, while *Lactiplantibacillus plantarum* and *Chryseobacterium carnipullorum* were significantly more prevalent in distant regions. The bacterial diversity does not have a clear relationship with the geographic proximity of farms, a finding that is consistent with the results of the principal coordinates analysis. Moreover, milk type had a pronounced impact on microbial diversity, with species such as *Acinetobacter johnsonii*, *Pseudomonas fragi*, *Enterobacter ludwigii*, *Achromobacter arsenitoxydans*, and *Leuconostoc mesenteroides* exhibiting substantial variations across different milk types. Furthermore, 22 species of lactic acid bacteria were identified in the studied milk samples. Notably, Holstein, donkey, and camel milk exhibited higher lactic acid bacteria diversity compared to buffalo and horse milk. Among these, *Lactiplantibacillus plantarum* was significantly more abundant in Holstein cow milk, while *Lactobacillus helveticus* and *Leuconostoc mesenteroides* were the predominant beneficial species in donkey and camel milk.

**IMPORTANCE:**

The data generated through this research present invaluable insights into the intricate microbial ecosystems inhabiting both Holstein and non-bovine milk varieties. These novel findings significantly advance our knowledge and approach toward understanding and managing microorganisms within milk, thereby bolstering their efficient utilization and facilitating the design of enhanced strategies for their prevention and control. This research not only sheds light on the diversity and dynamics of microbial communities in different milk types but also paves the way for more targeted interventions to ensure milk safety and quality across different production systems.

## INTRODUCTION

Milk contains nearly all the essential nutrients required by the human body, including proteins, fats, and minerals, which are vital for maintaining human health (1). Additionally, the nutrient-rich composition of raw milk provides ideal growth conditions, effectively serving as a natural culture medium for microorganisms (2). High-throughput sequencing analysis revealed that the predominant microorganisms in most raw milk samples were bacteria, with common genera including *Lactobacillus*, *Streptococcus*, *Enterococcus*, as well as psychrophilic bacteria such as *Pseudomonas* and *Fusobacterium* (3, 4). Among these, bacteria such as *Staphylococcus aureus*, *Escherichia coli*, *Klebsiella pneumoniae*, *Listeria monocytogenes*, and *Salmonella Typhimurium* have been identified as pathogens (5), while *Lactobacillus, Enterococcus, Streptococcus,* and *Lactococcus* are regarded as probiotic bacteria (6).

It is well-established that the microbial composition of milk is affected by various factors, including season, diet, region, and the species of dairy animals. Specifically, many previous studies investigated the variability of microorganisms in raw milk influenced by different regions. Bacterial diversity in cow milk at the genus level was compared in Moscow and Tula of Russia using Illumina sequencing (7). An investigation of bacterial genera in tank cow milk from two areas in Norway was performed (8). In southern Germany, the genus-level bacteria composition in raw cow milk during January was analyzed through Illumina sequencing (3). Moreover, significant differences in bacterial species were identified in raw cow milk across five provinces in China using PacBio single-molecule real-time (SMRT) sequencing (9). Illumina high-throughput sequencing technology was employed to compare the bacterial genera present in donkey milk samples collected from Xinjiang and Shandong provinces in China (10). Additionally, psychrophilic bacterial species composition was identified in raw cow milk collected from six regions in China through the method of cultivation coupled with shotgun metagenomic sequencing (11). The indigenous microbiota of non-bovine milk has also received increased attention in recent years, including buffalo milk (12), camel milk (13), and donkey milk (14), with significantly different dominant bacteria at the genus level. Furthermore, the total mesophilic aerobic bacteria, *Escherichia coli*, *Staphylococcus aureus,* and lactic acid bacteria counts in the Maghrebi camel were significantly higher than those in the *Gharbi* sheep and local goat milk from southwestern Tunisia using culture-based methods (15). Similarly, the quantities of *Enterobacter*, *Bacillus cereus*, *Listeria monocytogenes*, *Staphylococcus aureus,* and lactic acid bacteria in Slovak sheep and goat milk from April to September were compared using culture method (16). It has been indicated that, similar to geographical factors, the type of raw milk has been shown to affect bacterial composition. As previously reported, several studies have investigated the microbial communities present in raw milk samples collected from distinct geographic regions and different milk types, primarily focusing on the comparison of specific bacterial counts using traditional plate culturing methods. However, the influence of production site proximity and raw milk type on bacterial communities has received limited attention.

Traditional methods for detecting bacterial communities in milk and dairy products have typically relied on culture-based techniques combined with biochemical tests. With the advancement of high-throughput microbiome analyses based on long-read single-molecule real-time sequencing technologies, exemplified by the major platforms PacBio SMRT and Oxford Nanopore, this approach enables high-resolution taxonomic classification, even at the species level (17). SMRT sequencing technology has been extensively employed to detect bacterial communities in complex samples and has also been applied to assess bacterial populations in milk and dairy products (18, 19).

Both geographical location and the type of raw milk can lead to differences in the bacterial community of raw milk. In this study, samples of Holstein cow milk from adjacent and distant regions were collected to investigate whether the distance of geographical regions exerts a significant influence on the bacterial communities present in milk. Additionally, non-bovine milk samples were collected from regions geographically adjacent to the Holstein cow sampling sites to facilitate a comparative analysis of bacterial communities, thereby enabling the investigation of microbial compositional differences among distinct raw milk types originating from neighboring locations. The findings may provide valuable insights into the variations in bacterial communities in raw milk, influenced by farm location and milk source.

## RESULTS

### Physicochemical properties and bacterial counts of raw milk

In Holstein cow milk, the fat content ranged from 3.82 to 4.19%, with GD (Guangdong) exhibiting the highest levels and JN (Jinan) the lowest. The protein content ranged from 3.4 to 3.25%, with QD (Qingdao) having the highest levels and JN the lowest. The total bacterial count and somatic cell count were significantly higher in JN compared to other regions, at 4.63 log CFU/mL and 5.46 log cells/mL, respectively (Table S1). In non-bovine milk, G-SN (buffalo milk from Guangxi) has the highest, while S-LN (donkey milk from Shandong) has the lowest fat and protein contents. X-MN (horse milk from Xinjiang) exhibited the highest lactose content, whereas X-LT (camel milk from Xinjiang) had the lowest (Table S2).

### Sequencing data statistics

Following sequencing, the raw sequences from all samples were subjected to quality control using QIIME2 software. Initially, a total of 1,064,592 raw reads of 16S rRNA were obtained. After denoising and filtering, 1,062,915 valid 16S rRNA sequences and 1,043,409 high-quality 16S rRNA sequences were retained. Additionally, 7,720 operational taxonomic units (OTUs) were identified. Rarefaction curves (Fig. S1a) and Shannon curves (Fig. S1b) demonstrated that the sequencing depth was adequate to capture the majority of bacterial sequences, meeting the requirements for subsequent bioinformatics analysis.

### OTU cluster analysis of microbial species

Holstein cow milk samples from different regions shared only 81 OTUs, indicating a diverse microbial composition with distinct characteristics in each sample (Fig. S2a). In adjacent regions, the DY (Dongying) and QD samples exhibited the highest counts of unique OTUs, with 764 and 631, respectively. The WF (Weifang) and ZB (Zibo) samples followed with 408 and 321 unique OTUs, respectively. Conversely, the JN and YT (Yantai) samples displayed the lowest numbers of unique OTUs, with 71 and 58, respectively. Among distant regions, the GD and XJ samples displayed similar quantities of OTUs, with 141 and 170, respectively. These counts were slightly higher than those of the JN and YT samples but lower than those observed in the ZB samples.

Among the five different types of raw milk samples, a total of 141 OTUs were shared (Fig. S2b). Holstein cow milk exhibited the highest number of unique OTUs, totaling 4,829. In contrast, non-bovine milk samples generally displayed fewer OTUs. Among these, camel milk and donkey milk had the highest number of unique OTUs, with 164 and 130, respectively. Buffalo milk had 35 unique OTUs, while horse milk had the lowest, with only eight unique OTUs.

### Analysis of alpha diversity in bacterial communities

**Fig 1:**
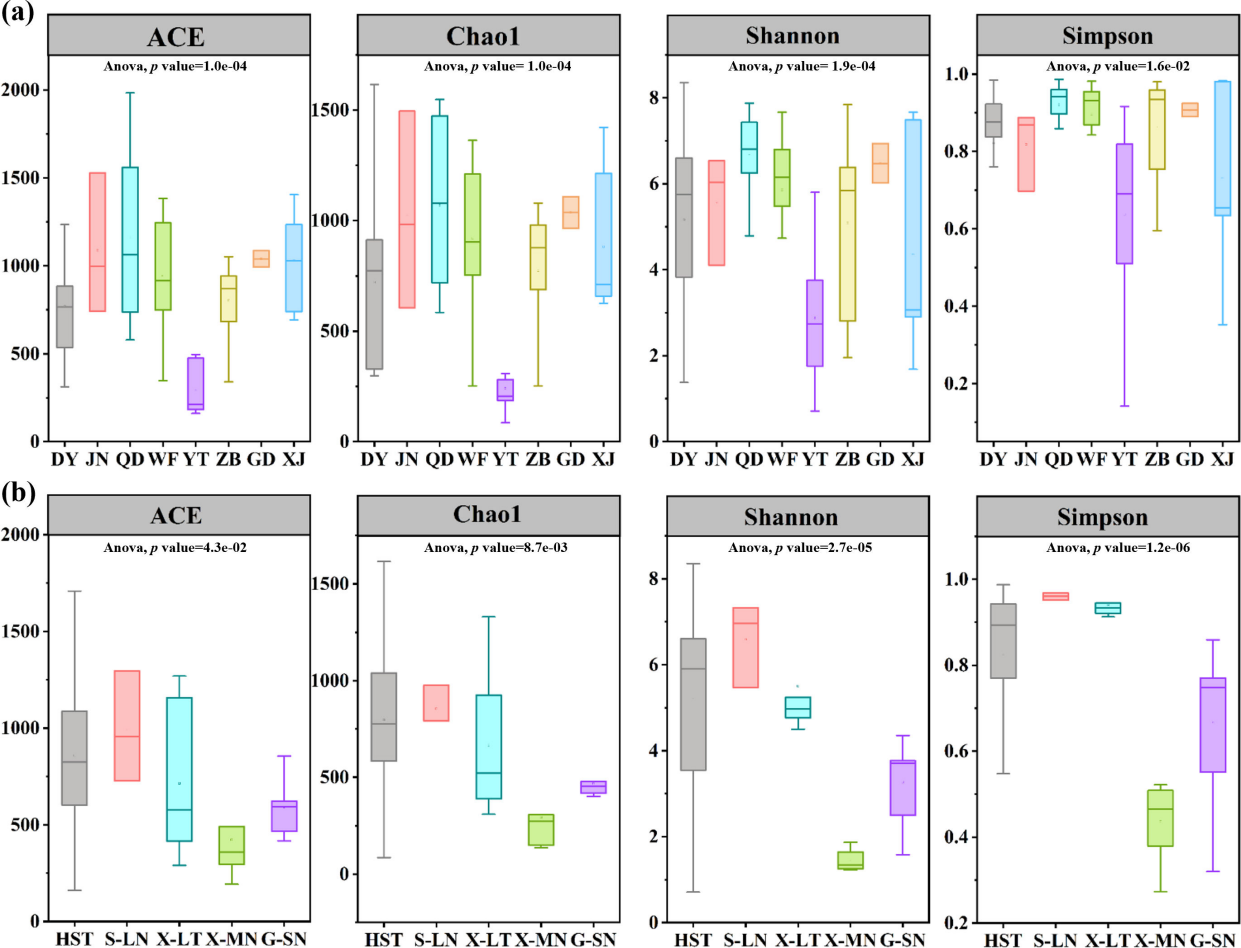
Alpha diversity index for different groups of raw milk samples from different regions (**a**) and types (**b**). Boxplots show the median (horizontal line), upper, and lower quartiles (top and bottom), 1.5 × interquartile range (whiskers). XJ, Holstein cow milk from Xinjiang; GD, Holstein cow milk from Guangdong; ZB, Holstein cow milk from Zibo; YT, Holstein cow milk from Yantai; JN, Holstein cow milk from Jinan; WF, Holstein cow milk from Weifang; QD, Holstein cow milk from Qingdao; DY, Holstein cow milk from Dongying; HST, Holstein cow milk; G-SN, buffalo milk from Guangxi; X-MN, horse milk from Xinjiang; X-LT, camel milk from Xinjiang; S-LN, donkey milk from Shandong.

Alpha diversity analysis provides insights into the richness and diversity of bacterial communities within individual samples. The richness of bacterial communities was assessed using the Chao1 and Abundance-based Coverage Estimator (ACE) indices, while diversity was evaluated using the Shannon and Simpson indices. For Holstein cow milk (Fig. 1a), in adjacent regions, QD samples exhibited the highest richness and diversity of bacterial communities, followed by JN, WF, ZB, and DY. In contrast, YT samples displayed significantly lower richness and diversity compared to the other regions. Among distant regions, no significant differences in bacterial community richness and diversity were observed between GD and XJ samples. Within these distant regions, GD samples exhibited higher richness and diversity, ranking second only to QD samples, while XJ samples demonstrated lower diversity, surpassing YT samples. Analyzing different types of raw milk (Fig. 1b), Holstein cow milk and donkey milk displayed the highest richness and diversity of bacterial communities, followed by camel milk and buffalo milk. Conversely, horse milk exhibited significantly lower richness and diversity compared to other types of milk.

### Analysis of beta diversity in bacterial communities

**Fig 2:**
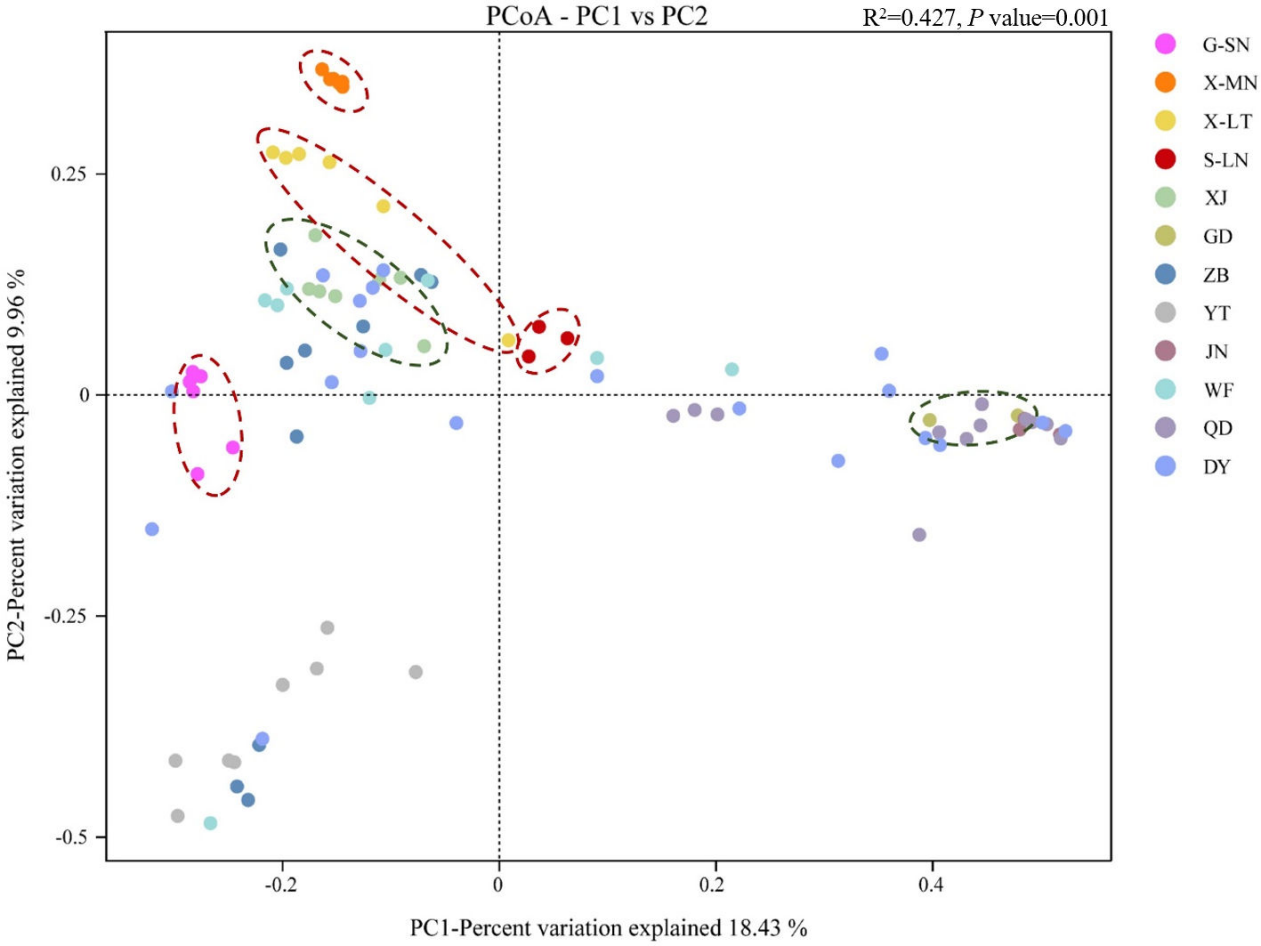
Principal coordinates analysis plots in groups of raw milk from different regions and types based on Bray-Curtis distance. G-SN, buffalo milk from Guangxi; X-MN, horse milk from Xinjiang; X-LT, camel milk from Xinjiang; S-LN, donkey milk from Shandong; XJ, Holstein cow milk from Xinjiang; GD, Holstein cow milk from Guangdong; ZB, Holstein cow milk from Zibo; YT, Holstein cow milk from Yantai; JN, Holstein cow milk from Jinan; WF, Holstein cow milk from Weifang; QD, Holstein cow milk from Qingdao; DY, Holstein cow milk from Dongying. Among them, the distribution ranges of non-bovine milk and distant Holstein cow milk are emphasized by red and green circular dotted frames, respectively.

The principal coordinate analysis of Holstein cow milk samples from diverse regions revealed distinct distribution patterns (Fig. 2). Within adjacent regions, JN and YT milk samples exhibited a clustering effect, indicating a higher similarity in bacterial community composition. In contrast, ZB, QD, WF, and DY samples showed more dispersed distributions. Notably, DY samples intersected with five other regions, while ZB and WF, as well as JN and QD samples, displayed some degree of clustering. However, ZB, QD, and YT samples were notably more distant from each other. Comparing the Holstein cow milk samples from distant regions, XJ and GD milk samples displayed tighter aggregation within their respective groups, which may be attributed to their smaller sample sizes. Nonetheless, a significant distance existed between XJ and GD samples in their distribution. Specifically, XJ samples clustered with ZB, WF, and DY samples, while being distinctly separated from YT, JN, and QD samples. Conversely, GD samples shared some similarities in microbial community composition with JN, QD, and DY samples, while differing more markedly from WF, ZB, and YT samples. In conclusion, variations in microbial community composition were evident among Holstein cow milk samples from different regions, but these differences did not strongly correlate with geographical distance.

Analysis of different types of raw milk revealed distinct clustering patterns, particularly evident within each non-bovine milk group, especially horse milk. Horse milk, camel milk, and XJ Holstein cow milk, originating from Xinjiang, predominantly clustered in the second quadrant. Despite this clustering, the distances separating these groups, especially between horse milk and XJ Holstein cow milk, were greater than the distances observed within each group. Conversely, donkey milk from Shandong exhibited a closer proximity to samples from WF, QD, and DY, while maintaining a significant distance from YT, JN, and ZB samples. Additionally, buffalo milk samples from Guangxi were distinctly different from GD samples but demonstrated a relatively closer association with DY samples. These findings indicate significant differences in microbial community composition among milk samples from different types, even within the adjacent regions.

### Analysis of the bacterial communities

To compare the diversity of microbial communities across various milk samples, species annotation was conducted on the microbial communities within each sample group. This annotation served as the foundation for subsequent analyses of intergroup differences in microbial compositions. Notably, despite variations in region or milk type, the predominant phyla across all samples were *Bacteroidetes*, *Proteobacteria*, and *Firmicutes*, albeit with varying relative abundances. Among the GD, QD, and JN Holstein cow milk samples, *Actinobacteria* exhibited the highest relative abundance, followed by *Firmicutes*, with *Proteobacteria* presenting a lower abundance. Conversely, in XJ Holstein cow milk, *Firmicutes* were most abundant, followed by *Proteobacteria*, while *Actinobacteria* had a lower relative abundance. For the remaining Holstein cow milk and non-bovine milk samples, *Proteobacteria* emerged as the dominant phylum, followed by *Firmicutes*, and with *Actinobacteria* presenting a lower relative abundance (Table S3).

The microbial composition at the genus level in Holstein cow milk samples varied significantly across different regions (Table S4). ZB samples were predominantly composed of *Acinetobacter* (20.33%), *Chryseobacterium* (12.59%), *Carnobacterium* (7.56%), and *Pseudomonas* (6.99%). In contrast, YT samples were dominated by *Pseudomonas* (44.84%), *Acinetobacter* (29.71%), *Chryseobacterium* (4.45%), and *Lactococcus* (3.52%). WF samples displayed a distinct microbial profile, with *Chryseobacterium* (12.07%), *Acinetobacter* (9.46%), *Streptococcus* (7.85%), and *Pseudomonas* (7.40%) as the prevailing genera. In QD and JN samples, *Lactiplantibacillus* (22.70% and 38.90%, respectively) and *unclassified Muribaculaceae* (5.21%, 5.23%) were the dominant genera. DY samples were characterized by *Pseudomonas* (11.67%), *Lactiplantibacillus* (10.83%), *Escherichia-Shigella* (7.84%), and *Chryseobacterium* (6.59%). Among distant regions, GD samples reflected a microbial composition similar to QD and JN, with *Lactiplantibacillus* (29.84%) and *unclassified Muribaculaceae* (4.87%) as the predominant genera. In contrast, XJ samples were distinguished by the dominance of *Chryseobacterium* (28.91%), *Escherichia-Shigella* (13.55%), *Streptococcus* (10.60%), and *Enhydrobacter* (6.70%).

**Fig 3:**
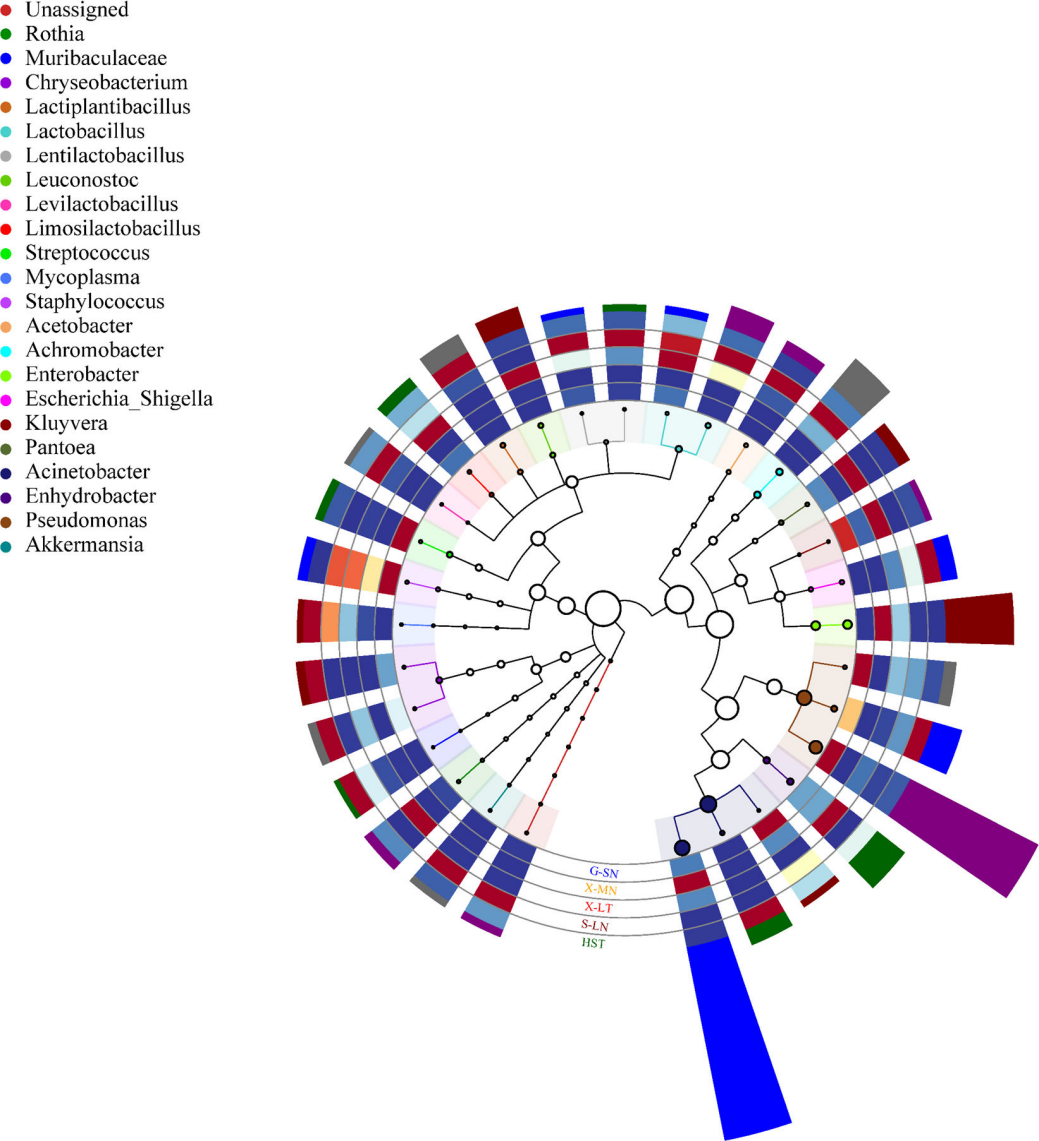
Dominant bacteria relative abundance at the genus level of different types of raw milk samples. HST, Holstein cow milk; G-SN, buffalo milk from Guangxi; X-MN, horse milk from Xinjiang; X-LT, camel milk from Xinjiang; S-LN, donkey milk from Shandong.

Significant differences in the predominant bacterial genera were also observed between Holstein cow milk and non-bovine milk in adjacent regions (Fig. 3). In Shandong, donkey milk was found to have high proportions of *Achromobacter* (11.65%), *Lactobacillus* (7.69%), and *Acetobacter* (5.89%), which contrasted sharply with the microbial composition of Holstein cow milk samples where these genera were either scarce or absent, particularly *Acetobacter*. Notably, *Carnobacterium*, a dominant genus in ZB Holstein cow milk samples, was absent in donkey milk. In Xinjiang, *Acinetobacter* was significantly elevated in both horse milk (69.75%) and camel milk (11.35%) compared to Holstein cow milk (1.42%). Additionally, horse milk exhibited a significant presence of *Enterobacter* (21.14%). Camel milk was dominated by *Leuconostoc* (9.30%), *Enhydrobacter* (8.70%), and *Pantoea* (7.29%), which were either minimally present or completely absent in Holstein cow milk. In Guangxi, buffalo milk was predominantly characterized by *Pseudomonas* (62.76%), *Acinetobacter* (11.10%), and *Streptococcus* (3.54%), in contrast to the GD Holstein cow milk samples, where these genera were present at negligible levels (<0.1%).

The bacterial classification was refined to the species level in this study (Table S5). ZB samples exhibited relatively higher abundances of *Acinetobacter albensis* (17.15%), *Carnobacterium maltaromaticum* (7.56%), *Pseudomonas lurida* (6.66%), and *Moraxella osloensis* (5.18%). In contrast, YT samples were dominated by *Pseudomonas lurida* (32.87%), *Acinetobacter albensis* (27.54%), and *Pseudomonas fragi* (11.78%) as prevalent species. WF samples displayed elevated abundances of *Haloanella gallinarum* (8.91%), *Acinetobacter albensis* (6.90%), *Pseudomonas lurida* (6.32%), and *Moraxella osloensis* (6.01%). QD and JN samples shared dominant species, including *Lactiplantibacillus plantarum* (22.70%, 38.90%), unclassified *Muribaculaceae* (5.21%, 5.23%), and uncultured *Bacteroidales bacterium* (2.78%, 2.98%). DY samples were primarily characterized by *Lactiplantibacillus plantarum* (10.83%), *Escherichia coli* (7.84%), and *Pseudomonas fragi* (6.47%). Among distant regions, GD samples had a similar profile to the QD and JN regions, with dominant species such as *Lactiplantibacillus plantarum* (29.84%), unclassified *Muribaculaceae* (4.87%), uncultured *Bacteroidales bacterium* (2.61%), and *Secundilactobacillus odoratitofui* (2.53%). In contrast, XJ samples were characterized by higher levels of *Chryseobacterium carnipullorum* (24.91%) and *Escherichia coli* (13.55%).

Significant variations in dominant bacterial species were observed among milk samples from different regions. The relative abundance of *Lactiplantibacillus plantarum* was significantly higher in JN and QD samples compared to ZB, YT, and WF samples. *Acinetobacter albensis* and *Pseudomonas lurida* exhibited notably higher relative abundances in YT samples than in other regions. Unclassified *Muribaculaceae* showed significantly higher abundances in QD and JN samples relative to other regions. *Haloanella gallinarum* was significantly more abundant in WF samples compared to YT, QD, and JN samples. Conversely, *Achromobacter arsenitoxydans* displayed a significantly lower abundance in YT samples compared to ZB and JN samples. Among distant regions, the relative abundance of *Lactiplantibacillus plantarum* was significantly lower in XJ samples compared to GD, whereas *Chryseobacterium carnipullorum* was notably higher in XJ than in other regions. Unclassified *Muribaculaceae* was significantly more abundant in GD samples than in XJ samples. Meanwhile, no significant differences in the relative abundances of *Escherichia coli*, *Moraxella osloensis*, and *Pseudomonas fragi* were observed across all samples.

Based on the relative abundance of microorganisms at the species level in different types of milk, camel milk exhibited significantly higher abundances of *Acinetobacter johnsonii* (9.41%), *Leuconostoc mesenteroides* (9.30%), and *Moraxella osloensis* (8.70%) compared to other milk types. Similarly, horse milk displayed notably elevated relative abundance of *Acinetobacter johnsonii* (69.51%) and *Enterobacter ludwigii* (21.14%). Additionally, horse milk was characterized by the presence of dominant species such as *Moraxella osloensis* (1.71%) and *Enterococcus faecalis* (1.28%). In Xinjiang, *Moraxella osloensis* emerged as a dominant species in horse milk, camel milk, and XJ Holstein cow milk. Both horse milk and XJ Holstein cow milk showed minimal abundances of *Pseudomonas fragi*, whereas camel milk had a relatively higher abundance (5.48%). Furthermore, significant differences were also observed in the relative abundances of other dominant species across the different milk types.

Donkey milk exhibited a significantly higher relative abundance of *Achromobacter arsenitoxydans* (11.65%), *Lactobacillus helveticus* (5.55%), and *Acetobacter pasteurianus* (5.30%) compared to other milk types, highlighting notable differences in dominant species relative to Holstein cow milk samples from Shandong. Conversely, buffalo milk was characterized by significantly higher abundances of *Pseudomonas fragi* (54.28%) and *Acinetobacter johnsonii* (8.22%) than other milks. Additionally, buffalo milk featured relatively elevated abundance of *Pseudomonas lurida* (4.76%) and *Pseudomonas laurentiana* (3.71%). In contrast to GD Holstein cow milk, where *Lactiplantibacillus plantarum* was the species with the highest relative abundance, buffalo milk was distinguished by the dominance of psychrophilic bacteria.

In terms of identifying bacterial species in milk, a total of 69 indigenous bacterial species (relative abundance >0.1%) were identified as shared among Holstein milk samples collected from six (ZB, YT, JN, WF, QD, DY). Among them, the greatest overlap in indigenous bacterial species was observed between QD and DY, which shared 106 species, whereas the lowest was recorded between YT and JN, with 78 shared taxa. In terms of distant regions, QD and GD exhibited the highest degree of taxonomic overlap, sharing 90 indigenous species, while QD and XJ shared the fewest, with only 67 species in common. Among different types of raw milk from adjacent regions, X-MN, X-LT, and XJ shared 54 indigenous bacteria, whereas G-SN and GD shared 76. Only 37 common indigenous bacteria were identified among different types of raw milk samples (Table S5).

A total of 22 lactic acid bacterial species were identified in both Holstein and non-bovine milk samples, each with a mean relative abundance greater than 0.1% (Table S6). Among these, Holstein cow milk exhibited the greatest diversity, with 22 lactic acid bacterial species being identified. In adjacent regions, *Lactiplantibacillus plantarum* was the most abundant in JN, QD, and DY samples, with relative abundances of 38.90%, 22.70%, and 10.83%, respectively, in stark contrast to its lower prevalence in ZB, WF, and YT samples. ZB samples, in particular, showed lower levels of lactic acid bacterial species, with *Lactobacillus helveticus* (1.98%) and *Limosilactobacillus pontis* (1.69%) being the most prominent. In both YT and WF samples, *Lactococcus piscium* was the predominant species, accounting for 2.68% and 2.28%, respectively. Additionally, *Lactobacillus johnsonii* (1.71%) and *Lactococcus lactis* (1.36%) were notably more prevalent in DY. In distant regions, *Lactiplantibacillus plantarum* dominated in GD samples, with a significantly higher relative abundance of 29.84%, similar to its dominance in JN, QD, and DY. Furthermore, *Levilactobacillus brevis* (1.35%) and *Lactobacillus vaccinostercus* DSM 20634 were also prevalent in GD. In XJ samples, the primary strains identified were *Streptococcus thermophilus* (1.78%) and *Lactococcus lactis* (1.03%). Based on these findings, the dominant lactic acid bacterial genera in Holstein cow milk were identified as *Lactobacillus* and *Lactococcus*.

Regarding non-bovine milk, donkey milk exhibited significantly higher diversity and relative abundance of lactic acid bacteria compared to other non-bovine milk types. In donkey milk, 20 lactic acid bacterial species were identified, with dominant species including *Lactobacillus helveticus* (5.55%), *Lentilactobacillus buchneri* (2.51%), and *Levilactobacillus brevis* (2.25%). Camel milk featured 18 beneficial species, with *Leuconostoc mesenteroides* (9.30%), *Limosilactobacillus pontis* (3.25%), and *Lactobacillus helveticus* (2.89%) as the predominant species. Eighteen lactic acid bacterial species were also identified in buffalo milk, while horse milk exhibited the lowest diversity and relative abundance of lactic acid bacteria, with only 16 species identified.

### Metabolic functions of microbial genes

A total of 317 Kyoto Encyclopedia of Genes and Genomes (KEGG) tertiary pathways were annotated across the different samples. The top 10 most abundant functional pathways were presented in Tables S7 and S8. Based on the analysis of Holstein cow milk samples from adjacent regions, YT exhibited the significantly highest levels in diverse environments, ABC transporters, and two-component systems. Carbon metabolism was significantly higher in ZB relative to other regions, while microbial metabolism in diverse environments in QD and ABC transporters in ZB was significantly lower than in other samples. From distant regions, biosynthesis of amino acids, quorum sensing, and carbon metabolism in GD showed significant differences from YT. For XJ, biosynthesis of secondary metabolites, carbon metabolism, ribosome, purine metabolism, and pyrimidine metabolism were significantly higher, whereas ABC transporters and two-component systems were significantly lower compared to YT.

Based on various types of milk, the biosynthesis of secondary metabolites, b1iosynthesis of antibiotics, biosynthesis of amino acids, and ribosome pathways were significantly lower, while the two-component system was significantly higher in G-SN compared to others. In X-MN, the biosynthesis of antibiotics, ABC transporters, and purine metabolism pathways exhibited significantly higher, while metabolic pathways and microbial metabolism in diverse environments were significantly lower compared to other milk types. It indicated that differences in bacterial composition in raw milk from different regions and types could relate to variations in milk metabolites.

### Correlation analysis of physicochemical indicators and dominant bacteria in non-bovine milk

**Fig 4:**
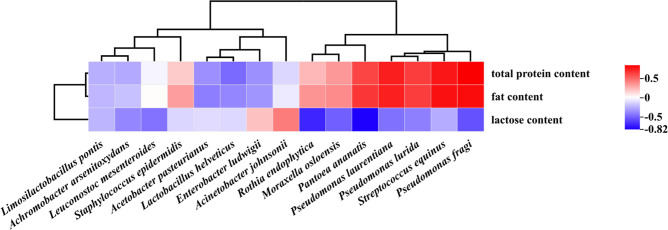
Correlation analysis of physicochemical indicators and dominant bacteria in non-bovine milk.

The fat, protein, and lactose contents of horse, camel, donkey, and buffalo milk were correlated with the relative abundance of the top 15 bacterial species through Spearman correlation analysis (Fig. 4). The fat and protein contents were found to be significantly and positively correlated with *Pseudomonas fragi* (ρ = 0.806, 0.846), *Pseudomonas laurentiana* (ρ = 0.746, 0.747), and *Streptococcus equinus* (ρ = 0.795, 0.773), while the lactose content was negatively correlated with *Pantoea ananatis* (ρ = −0.821) and *Rothia endophytica* (ρ = −0.702).

## DISCUSSION

The relative abundance of microbial communities indicated that *Acinetobacter*, *Pseudomonas*, and *Lactiplantibacillus* had relatively high abundance at the genus level in Holstein cow milk. This finding is consistent with the predominant genus composition observed in Holstein cow milk from three farms across different regions of Poland (20). Similarly, 16S rRNA gene sequencing results revealed that *Bacillus*, *Pseudomonas*, *Streptococcus*, *Lactococcus*, and *Acinetobacter* were the dominant genera in raw milk samples collected from two dairy plants in different geographical locations in Norway (21). Correspondingly, at the species level, *Lactiplantibacillus plantarum*, *Pseudomonas lurida*, and *Acinetobacter albensis* exhibited relatively higher relative abundance. In contrast, raw milk samples from Beijing, Hebei, Inner Mongolia, Shanghai, and Guangdong in China were investigated using SMRT technology and identified *Acinetobacter albensis*, *Pseudomonas gessardii*, *Pseudomonas weihenstephanensis*, and *Rahnella inusitata* as the most abundant bacteria (9). These suggest that the genus-level microbial composition is similar in raw milk across different regions, while there are greater differences at the species level. These differences do not appear to be strongly correlated with geographic proximity of farms, but rather may be influenced by factors such as the feeding environment and management practices (22).

Alpha diversity analysis revealed that microbial diversity in bovine milk samples is significantly higher compared to non-bovine milk samples, a pattern also observed in other studies (23, 24). In this study, the dominant bacterial genera in buffalo milk were identified as *Acinetobacter*, *Pseudomonas*, and *Streptococcus*, consistent with the core microbial community described for buffalo milk in Guangxi (China) (25). For camel milk, the most abundant genera were *Acinetobacter*, *Pseudomonas*, *Enterobacter*, and *Enhydrobacter*. Similarly, *Acinetobacter* and *Pseudomonas* were noted as the core microbial genera in raw camel milk from Kuwait (26). However, a higher presence of *Lactobacillus*, *Enterococcus*, and *Streptococcus* in camel milk was noted in other research, albeit in lower relative abundance than in this study. In contrast, horse milk from the Xilingol League in the Inner Mongolia Autonomous Region was reported to predominantly feature *Lelliottia* and *Pseudomonas* (27), whereas our study identified *Acinetobacter* and *Enterobacter* as the dominant genera. Donkey milk has been reported in Cyprus to be primarily composed of gram-negative pleomorphic bacteria, including *Sphingomonas*, *Pseudomonas*, *Mesorhizobium*, *Acinetobacter*, *Lactobacillus*, *Streptococcus*, *Clostridium*, *Flavobacterium*, and *Ralstonia* (28). In the present study, most of these taxa were detected; however, *Flavobacterium* and *Ralstonia* were absent. Significant differences in dominant bacterial species were observed across non-bovine milk types: *Acinetobacter johnsonii* and *Enterobacter ludwigii* were prevalent in horse milk; *Pseudomonas fragi* and *Acinetobacter johnsonii* were prevalent in buffalo milk; *Acinetobacter johnsonii*, *Leuconostoc mesenteroides*, and *Moraxella osloensis* were noted in camel milk; and *Achromobacter arsenitoxydans* and *Lactobacillus helveticus* were identified in donkey milk. Overall, significant differences in microbial composition and relative abundance exist between Holstein and non-bovine milks. The type of milk appears to have a more significant impact on microbial composition than geographic proximity of farms.

Buffalos are commonly raised in moist environments such as lakes, rivers, and wetlands, which increases their susceptibility to psychrotrophic bacteria, including *Pseudomonas* and *Fusobacterium*. These bacteria can proliferate in buffalo milk, leading to a higher relative abundance of these microorganisms. Horse and donkey milk, which have higher lactose content (29), facilitate the growth of *Enterobacter* due to the presence of lactase enzyme that breaks down lactose, allowing it to be used as a carbon source for bacterial growth. Consequently, *Enterobacter*, particularly *Enterobacter ludwigii*, was found to be more abundant in horse and donkey milk in our studies. In contrast, lower fat content in horse milk resulted in less energy available to support bacterial growth. Otherwise, a higher proportion of polyunsaturated fatty acids in horse milk (30) may influence the stability and permeability of bacterial cell membranes, potentially contributing to the lower bacterial abundance observed in horse milk. Camel milk is characterized by a higher lactoferrin content, which imparts stronger antimicrobial activity (31). Additionally, whey proteins in camel milk have been shown to significantly inhibit the growth of pathogenic bacteria such as *Escherichia coli* and *Listeria monocytogenes* (32). Consequently, camel milk generally has a lower relative abundance of bacteria. Overall, non-bovine milks, particularly horse and buffalo milk, have shown a higher relative abundance of psychrotrophic bacteria compared to Holstein cow milk. This suggests that non-bovine milks may pose a greater food safety risk. This finding was also reported in the previous study (23).

A total of 22 lactic acid bacterial species were shared across all samples in this study. Holstein cow milk contained the highest number of lactic acid bacterial species, with 22 species identified, aligning with previous research findings (33). *Lactobacillus plantarum* was identified in all samples, with the highest relative abundance observed in Holstein cow milk, particularly in the JN, QD, GD, and DY samples. This may be related to the feeds, as fermented feeds have been shown to elevate *Lactobacillus* levels in the intestines (34), potentially enhancing the quality of raw milk. Recent studies have consistently identified *Lactobacillus plantarum* in both Holstein and camel milk. For instance, 4 out of 16 strains of *Lactobacillus plantarum* were found in the lactic acid bacterial community from camel milk in Egypt (35). Twenty-seven strains of *Lactobacillus plantarum*, including 16 strains of *Lactiplantibacillus plantarum*, were isolated from fresh Holstein cow milk collected from domesticated Holsteins in Yongdong, Korea (36). *Lactobacillus plantarum* is known for its beneficial biological activities, such as stimulating the immune system, inhibiting the growth of pathogens, and regulating intestinal microbiota balance (37). Metabolomics studies on *Lactobacillus plantarum* strain NMGL2, isolated from traditional Inner Mongolian cheese, revealed its ability to enhance cell wall component biosynthesis and activate the glutamic acid decarboxylation system (38). This strain also exhibited significant cold resistance and acid tolerance, producing volatile compounds such as acetic acid, valeric acid, and heptanoic acid, which are essential in developing the unique flavor profile of natural fermented milk.

Lactose, the predominant carbohydrate in milk, serves as a vital nutritional substrate for microorganisms. Variations in lactose content among different raw milk types highlight its significance as a quality indicator, given its stable levels under compatible milk production conditions. Correlation analyses indicated a negative relationship between lactose and most bacteria in non-bovine milk. This key carbon source undergoes glycolytic breakdown into glucose by various bacteria (39, 40). Previous research has demonstrated the lactose-reducing capabilities of *Enterobacter* and *Actinomyces* in human breast milk during lactation (41). Actinomycetes have been observed to decrease lactose levels in human breast milk during lactation. The total bacterial count has shown a negative correlation with lactose content in bulk-tank milk sourced from Brazil (42).

### Conclusion

The research identified significant variations in the microbial composition of Holstein cow milk across different farms, with notable differences observed at the species level for *Lactobacillus plantarum* and *Chryseobacterium carnipullorum*. These variations were not closely linked to the geographic proximity of the farms, suggesting that underlying environmental factors at the farm level may substantially influence microbial diversity. The study also highlighted the impact of milk type on microbial diversity from adjacent regions, with prominent species such as *Acinetobacter johnsonii*, *Pseudomonas fragi*, *Enterobacter ludwigii*, *Achromobacter arsenitoxydans*, and *Leuconostoc mesenteroides* being identified. This suggested that the milk matrix may have a greater influence on bacterial community composition. Additionally, lactic acid bacteria species like *Lactobacillus plantarum*, *Lactobacillus helveticus*, and *Leuconostoc mesenteroides* were found across the studied milk types. Considering the influence of sample type and quantity, future experiments will involve an expanded collection of both Holstein cow and non-bovine milk samples from a wider range of geographical regions. This will allow for a comparative analysis of the bacterial community composition between bovine and non-bovine milk from adjacent areas, as well as among samples of the same milk type collected from geographically distant locations. Such an approach will enhance the robustness of evaluating the respective contributions of milk type and geographical origin to microbial diversity in raw milk.

## MATERIALS AND METHODS

### Sample collection

Dairy farms in China are distributed across a vast geographic area. In this study, dairy farms with over 500 Holstein cows were selected from Shandong, Guangdong, and Xinjiang, located in the eastern, southern, and western parts of China, respectively. A total of 32 dairy farms from Shandong were included, with the farms distributed across five regions: ZB with five farms, YT with four farms, WF with five farms, QD with six farms, JN with two farms, and DY with 10 farms. These areas in Shandong were classified as adjacent regions. Additionally, two dairy farms from Guangdong and three dairy farms from Xinjiang, which are geographically distant from Shandong, were classified as distant regions. From each dairy farm, two bulk-tank milk samples were collected on separate days and stored in sterile bottles at −20°C until further analysis. In addition to Holstein cows, samples were also collected from other types of dairy farms: two donkey farms from Shandong, three buffalo farms from Guangxi, three camel farms, and three horse farms from Xinjiang (Fig. S3). Two samples were obtained from each of these farms, following the same storage protocol. All the raw milk samples were collected in September–October 2022.

### Analysis of raw milk components and determination of bacterial counts

Milk samples were analyzed using a MilkoScan FT120 analyzer (Foss Electric, Denmark) to determine fat, protein, and lactose contents, while somatic cell count was measured using a Fossomatic 5000 (Foss Electric) (43).

For the determination of titratable acidity, 10 g of each milk sample was mixed with 20 mL of distilled water. The resulting mixture was titrated using a 0.1 mol/L NaOH solution, with the addition of 2 mL of 0.5% (vol/vol) phenolphthalein as an indicator. The titration process was continued until a faint pink color appeared, indicating the endpoint of the titration. One sample from each batch was subjected to triplicate analysis (44).

For microbiological analysis, total bacterial counts were determined by plate counting. Each milk sample was serially diluted by adding 1 mL of sample to 9 mL of sterile physiological saline (0.85% NaCl, wt/vol) until a final dilution of 10^−6^ was achieved. One milliliter aliquot of each diluted sample was then used to determine colony counts using the pour plate method on plate count agar. Agar plates were incubated at 37°C for 36 to 48 h, after which only plates containing 30 to 300 colonies were selected for counting. The results were expressed as colony-forming units per milliliter of milk. All experiments were performed in triplicate to ensure accuracy and reproducibility.

### DNA extraction

Total genomic DNA was extracted from raw milk samples using the TGuide S96 Magnetic Soil/Stool DNA Kit (Tiangen Biotech [Beijing] Co., Ltd.) according to the manufacturer’s instructions. The extracted DNA samples were then stored at −20°C for further analysis.

### Quality analysis of DNA

The concentration and purity of DNA were assessed using a spectrophotometer (NanoDrop 2000 UV-Vis, Thermo Scientific, Wilmington, USA). The quality and quantity of the extracted DNA were further verified by electrophoresis on a 1.8% agarose gel. The DNA was diluted to 1 ng/µL for use as a PCR template. Following the DNA quality assessment, it was observed that seven samples exhibited suboptimal DNA quality. These included one DY sample, one JN sample, one WF sample, two GD samples, one XJ sample, and one LN sample.

### PCR amplification, purification, and single-molecule real-time sequencing

The forward 27F (5´–3´): AGRGTTTGATYNTGGCTCAG and reverse 1492R (5´–3´): TASGGHTACCTTGTTASGACTT primers were employed to amplify the extracted DNA. Both primers were tailed with sample-specific PacBio barcode sequences to facilitate multiplexed sequencing. The use of barcoded primers was chosen to minimize chimera formation, compared to protocols where primers are added in a subsequent PCR reaction.

PCR amplification was carried out using the KOD One PCR Master Mix (Toyobo Life Science) across 25 cycles. The amplification protocol included an initial denaturation at 95°C for 2 min, followed by 25 cycles of denaturation at 98°C for 10 s, annealing at 55°C for 30 s, and extension at 72°C for 1 min and 30 s, concluding with a final extension at 72°C for 2 min. The PCR amplicons were purified using VAHTS DNA Clean Beads (Vazyme, Nanjing, China) and quantified with the Qubit dsDNA HS Assay Kit and Qubit 3.0 Fluorometer (Invitrogen, Thermo Fisher Scientific, Oregon, USA). Following individual quantification, the amplicons were pooled in equal amounts.

SMRTbell libraries were prepared from the amplified DNA using the SMRTbell Express Template Prep Kit 2.0 according to the manufacturer’s instructions (Pacific Biosciences). The purified SMRTbell libraries from the pooled and barcoded samples were sequenced on a PacBio Sequel II platform (Beijing Biomarker Technologies Co., Ltd., Beijing, China) utilizing the Sequel II Binding Kit 2.0.

### Bioinformatics processing and statistical analysis

The raw 16S rRNA sequencing data (BioProject No. PRJNA1294854 in the NCBI database) were initially subjected to quality filtering using Trimmomatic (version 0.33). Subsequently, primer sequences were identified and removed with Cutadapt (version 1.9.1). Following this, paired-end reads were spliced and chimeras were removed using USEARCH (version 10) and UCHIME (version 8.1) to obtain high-quality sequences. Sequences were clustered at a default similarity threshold of 97% with USEARCH (version 10.0), and OTUs were filtered using a threshold of 0.005% of the total number of sequenced reads. According to previous reports, this clustering strategy effectively minimized the impact of sequencing errors, thereby enhancing the accuracy and robustness of taxonomic assignments (45). Moreover, it reduced excessive fragmentation of microbial communities and facilitated a more consistent and reliable characterization of community structures, particularly at the genus and species levels (46).

Alpha diversity and beta diversity indices were assessed using QIIME2 software. Beta diversity analysis was conducted using the Bray-Curtis algorithm to calculate the distances between samples, thereby obtaining beta diversity values.

The feature sequences were taxonomically annotated using the naive Bayesian classifier in conjunction with alignment against the SILVA reference database, thereby providing species classification information for each feature. Following this, the microbial community composition in each sample was quantified at various taxonomic levels (phylum, genus, species), and an abundance table at different classification levels was generated using QIIME software.

16S rRNA was aligned to reference sequences from the Integrated Microbial Genomes database to construct a phylogenetic tree with PICRUSt2. This process identified the “nearest species” of the feature sequences. Genetic information for unknown species was inferred based on known gene categories and abundance data, enabling the prediction of metabolic pathways for the entire community in conjunction with KEGG pathway information.

Graphic presentations of bacterial diversity were performed with the aid of the BMKCloud (http://www.biocloud.net/). Analysis of variance was performed using SPSS software to assess the significance of alpha diversity indices, relative abundance of dominant bacterial species, and KEGG pathway among different samples. Correlations between physicochemical indicators and bacteria in non-bovine milk were examined using Spearman correlation analysis (ρ > 0.6, *P* < 0.05) performed in IBM SPSS Statistics (version 27.0.1) and subsequently visualized with Origin (version 64).

## Data Availability

All raw 16S rRNA gene sequencing data have been deposited in the Sequence Read Archive (SRA) of NCBI under the BioProject accession number PRJNA1294854.

## SUPPLEMENTAL MATERIAL

Figures S1 to S3 spectrum.00933-25-s0001.docx Fig. S1: Rarefaction curves (a) and Shannon curves (b) of the bacterial communities for each group from raw milks. Fig. S2: Upset diagram of operational taxonomic units of bacteria at amplicon sequence variants level in raw milk from different regions (a) and types (b). Fig. S3: Collecting regions of Holstein cow milk and non-bovine milk.

Table S1 spectrum.00933-25-s0002.docx Differences in physicochemical properties of Holstein cow milk from different regions.

Table S2 spectrum.00933-25-s0003.docx Differences in physicochemical properties of raw milk from different types.

Table S3 spectrum.00933-25-s0004.docx Relative abundance of bacteria at the phylum level of raw milk from different regions and types (>0.1%).

Table S4 spectrum.00933-25-s0005.docx Relative abundance of bacteria at the genus level of raw milk from different regions and types (>0.1).

Table S5 spectrum.00933-25-s0006.docx Relative abundance of bacteria at the species level of raw milk from different regions and types (>0.1%).

Table S6 spectrum.00933-25-s0007.docx Relative abundance of lactic acid bacteria at the species level of raw milk from different regions and types (>0.1%).

Table S7 spectrum.00933-25-s0008.xlsx Comparation of KEGG tertiary pathways between Holstein cow milk from different regions (top 10).

Table S8 spectrum.00933-25-s0009.xlsx Comparation of KEGG tertiary pathways between raw milk from different types (top 10).

ASM does not own the copyrights to Supplemental Material that may be linked to, or accessed through, an article. The authors have granted ASM a non-exclusive, world-wide license to publish the Supplemental Material files. Please contact the corresponding author directly for reuse.
