# Supplementary material for: Mapping bacterial microbiota variations in raw milk: geographic and type-specific insights
Source: Microbiol Spectr. 2025 Oct 27;13(12):e00933-25. doi: 10.1128/spectrum.00933-25 (PMC12671074; doi:10.1128/spectrum.00933-25)
Supplement: Figures S1 to S3 — Fig. S1: Rarefaction curves (a) and Shannon curves (b) of the bacterial communities for each group from raw milks. Fig. S2: Upset diagram of operational taxonomic units of bacteria at amplicon sequence variants level in raw milk from different regions (a) and types (b). Fig. S3: Collecting regions of Holstein cow milk and non-bovine milk. [file spectrum.00933-25-s0001.docx]

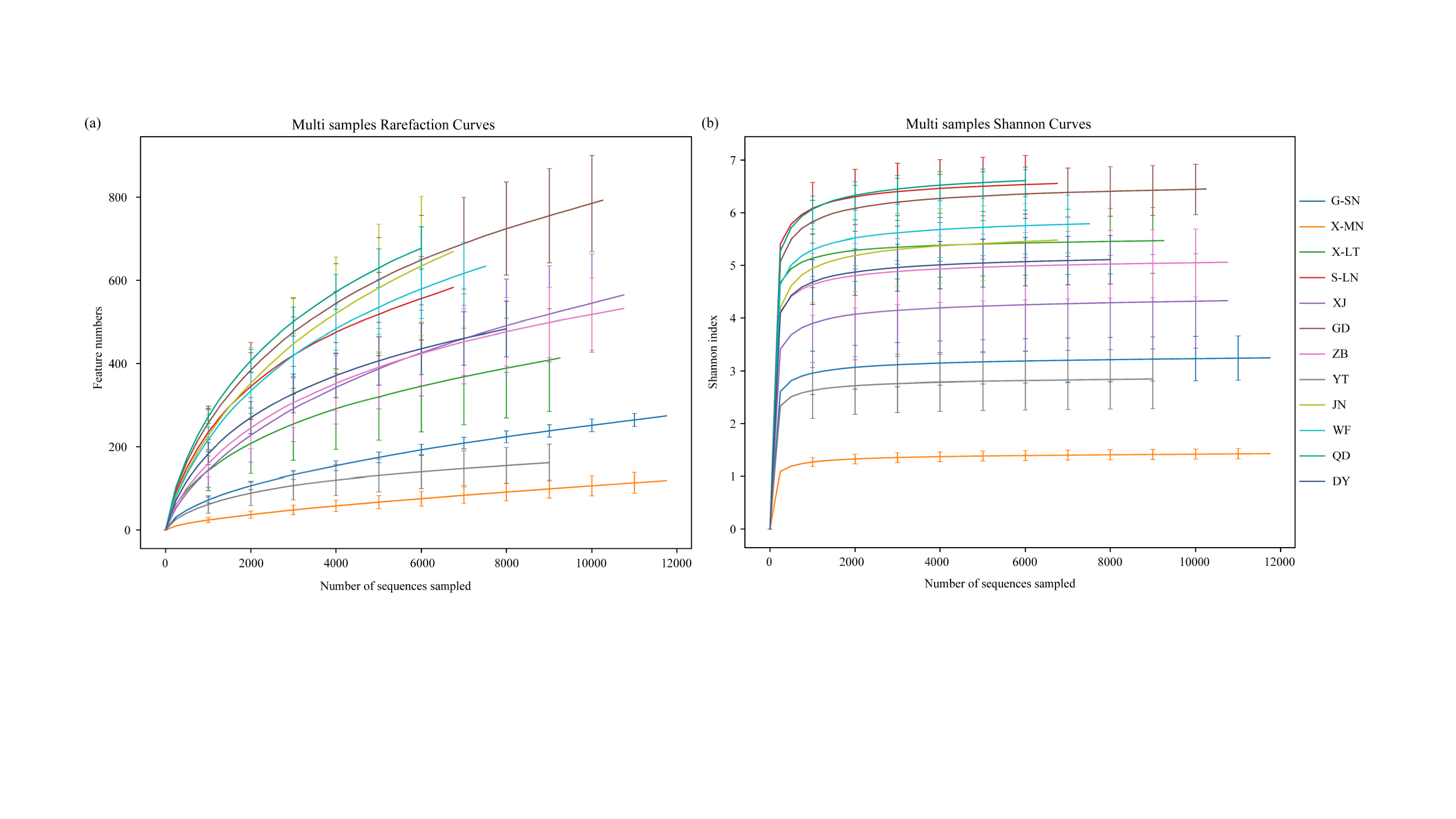
Fig. S1 Rarefaction curves (a) and Shannon curves (b) of the bacterial communities for each group from raw milks.

Note: G-SN, buffalo milk from Guangxi; X-MN, horse milk from Xingjiang; X-LT, camel milk from Xinjiang; S-LN, donkey milk from Shandong; XJ, Holstein cow milk from Xinjiang; GD, Holstein cow milk from Guangdong; ZB, Holstein cow milk from Zibo; YT, Holstein cow milk from Yantai; JN, Holstein cow milk from Jinan; WF, Holstein cow milk from Weifang; QD, Holstein cow milk from Qingdao; DY, Holstein cow milk from Dongying.


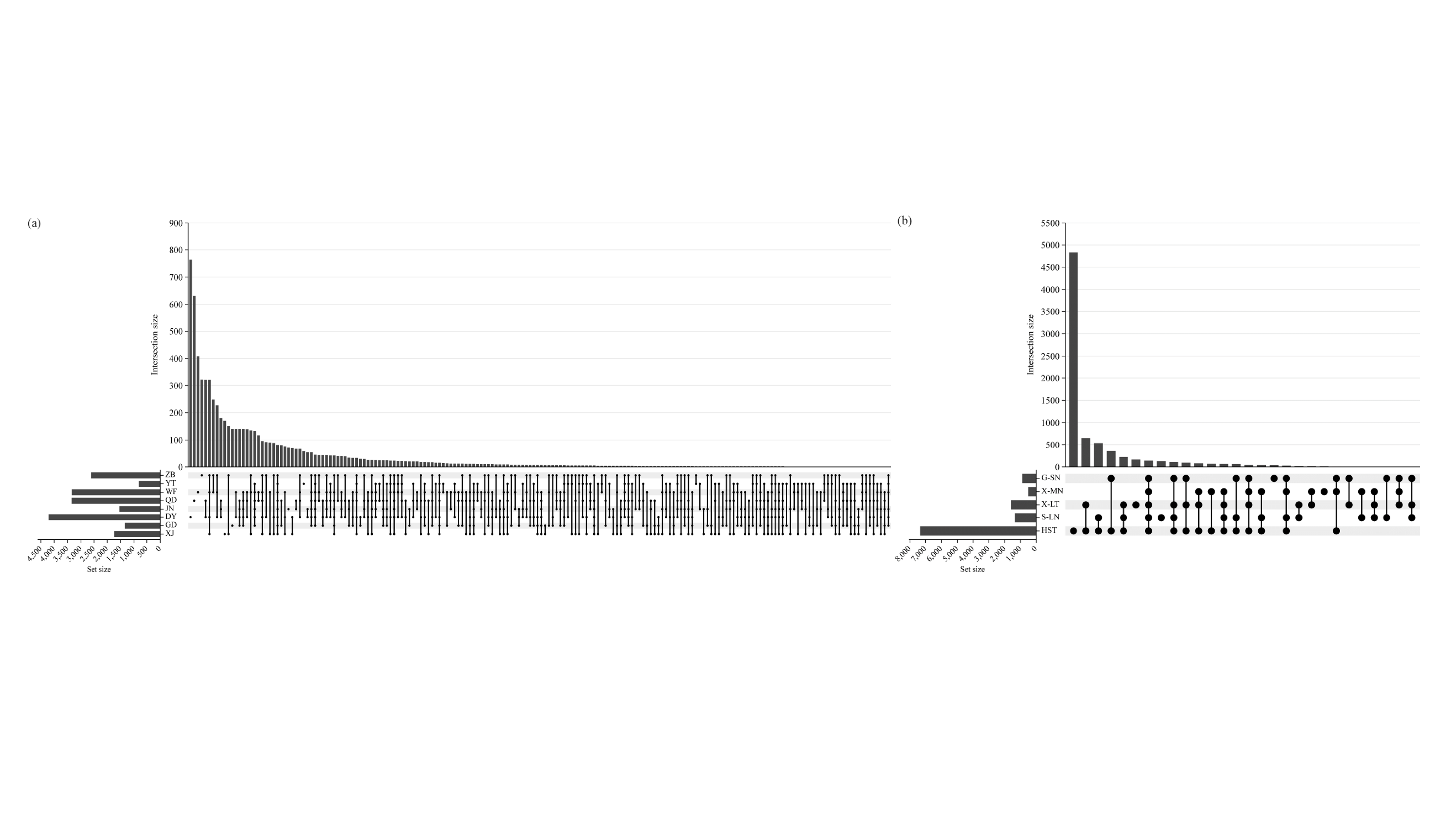
Fig. S2 Upset diagram of operational taxonomic units of bacteria at amplicon sequence variants level in raw milk from different regions (a) and types(b).

Note: G-SN, buffalo milk from Guangxi; X-MN, horse milk from Xingjiang; X-LT, camel milk from Xinjiang; S-LN, donkey milk from Shandong; XJ, Holstein cow milk from Xinjiang; GD, Holstein cow milk from Guangdong; ZB, Holstein cow milk from Zibo; YT, Holstein cow milk from Yantai; JN, Holstein cow milk from Jinan; WF, Holstein cow milk from Weifang; QD, Holstein cow milk from Qingdao; DY, Holstein cow milk from Dongying.


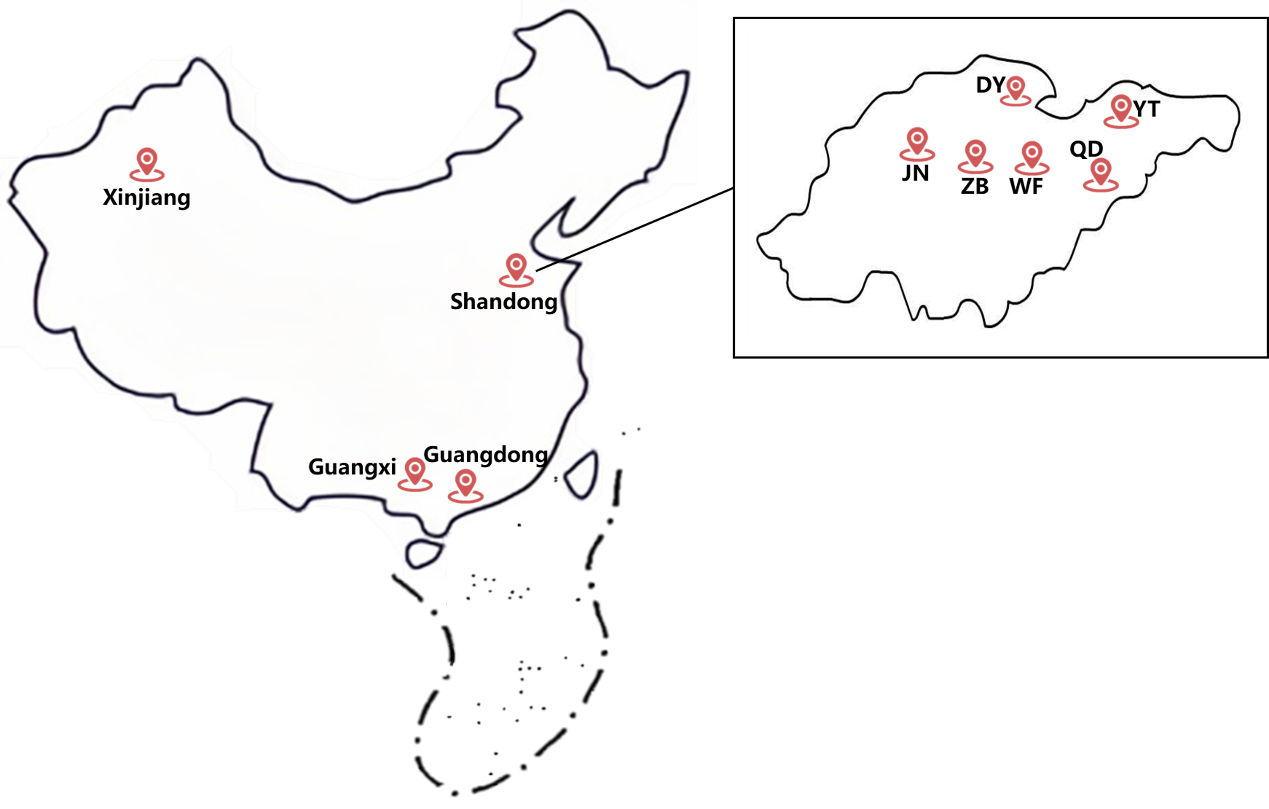
Fig. S3 Collecting regions of Holstein cow milk and non-bovine milk.

Note: ZB, Zibo; YT, Yantai; JN, Jinan; WF, Weifang; QD, Qingdao; DY, Dongying.
