# Supplementary material for: Mapping bacterial microbiota variations in raw milk: geographic and type-specific insights
Source: Microbiol Spectr. 2025 Oct 27;13(12):e00933-25. doi: 10.1128/spectrum.00933-25 (PMC12671074; doi:10.1128/spectrum.00933-25)
Supplement: Table S1 — Differences in physicochemical properties of Holstein cow milk from different regions. [file spectrum.00933-25-s0002.docx]

Table S1 Differences in physicochemical properties of Holstein cow milk from different regions.

| Samples | Titratable acidity | Fat contents (%) | Protein contents (%) | Total bacterial count (×10^4^ CFU/mL) | Somatic cell count (×10^4^ cells/mL) |
| --- | --- | --- | --- | --- | --- |
| ZB | 13.34±0.27 | 3.98±0.11abc | 3.32±0.05bcd | 2.22±1.58b | 19.35±8.00b |
| YT | 13.35±0.10 | 4.01±0.13abc | 3.37±0.05ab | 2.63±1.83b | 21.19±5.41b |
| WF | 13.44±0.18 | 3.94±0.16bc | 3.32±0.06bcd | 1.89±0.86b | 21.36±2.76b |
| QD | 13.38±0.39 | 3.93±0.24bc | 3.40±0.09a | 2.39±1.63b | 21.55±7.38b |
| JN | 13.48±0.15 | 3.82±0.10c | 3.25±0.12d | 4.30±2.34a | 29.08±7.14a |
| DY | 13.44±0.28 | 4.06±0.22ab | 3.33±0.06abc | 2.07±1.30b | 20.49±6.19b |
| GD | 13.52±0.25 | 4.19±0.28a | 3.32±0.06bcd | 1.72±1.09b | 23.79±3.38ab |
| XJ | 13.34±0.28 | 4.08±0.19ab | 3.28±0.10cd | 2.03±1.60b | 22.39±5.11b |

Note: XJ, Holstein cow milk from Xinjiang; GD, Holstein cow milk from Guangdong; ZB, Holstein cow milk from Zibo; YT, Holstein cow milk from Yantai; JN, Holstein cow milk from Jinan; WF, Holstein cow milk from Weifang; QD, Holstein cow milk from Qingdao; DY, Holstein cow milk from Dongying. a-d means within a column with different superscripts are significantly different (*P* < 0.05).
