# Supplementary material for: Mapping bacterial microbiota variations in raw milk: geographic and type-specific insights
Source: Microbiol Spectr. 2025 Oct 27;13(12):e00933-25. doi: 10.1128/spectrum.00933-25 (PMC12671074; doi:10.1128/spectrum.00933-25)
Supplement: Table S2 — Differences in physicochemical properties of raw milk from different types. [file spectrum.00933-25-s0003.docx]

Table S2 Differences in physicochemical properties of raw milk from different types.

| Samples | Fat contents (%) | Protein contents (%) | Lactose contents (%) |
| --- | --- | --- | --- |
| X-MN | 1.17±0.07c | 1.95±0.08c | 6.52±0.18a |
| S-LN | 0.47±0.15d | 1.58±0.16d | 6.02±0.27b |
| X-LT | 5.75±0.34b | 3.76±0.28b | 4.94±0.28d |
| G-SN | 7.83±0.22a | 4.67±0.31a | 5.60±0.10c |

Note: G-SN, buffalo milk from Guangxi; X-MN, horse milk from Xingjiang; X-LT, camel milk from Xinjiang; S-LN, donkey milk from Shandong. a-d means within a column with different superscripts are significantly different (*P* < 0.05).
