# Supplementary material for: Mapping bacterial microbiota variations in raw milk: geographic and type-specific insights
Source: Microbiol Spectr. 2025 Oct 27;13(12):e00933-25. doi: 10.1128/spectrum.00933-25 (PMC12671074; doi:10.1128/spectrum.00933-25)
Supplement: Table S3 — Relative abundance of bacteria at the phylum level of raw milk from different regions and types (>0.1%). [file spectrum.00933-25-s0004.docx]

| phylum | G-SN* (%) | X-MN* (%) | X-LT* (%) | S-LN* (%) | XJ* (%) | GD* (%) | ZB* (%) | YT* (%) | JN* (%) | WF* (%) | QD* (%) | DY* (%) |
| --- | --- | --- | --- | --- | --- | --- | --- | --- | --- | --- | --- | --- |
| *Proteobacteria* | 81.51 | 93.72 | 55.51 | 41.37 | 30.31 | 17.07 | 44.62 | 81.58 | 15.00 | 38.06 | 19.92 | 39.29 |
| *Firmicutes* | 10.96 | 4.73 | 27.36 | 35.00 | 28.54 | 49.80 | 25.82 | 11.08 | 59.97 | 30.14 | 48.60 | 32.46 |
| *Bacteroidota* | 5.58 | 0.73 | 3.65 | 6.54 | 34.99 | 25.16 | 22.98 | 5.98 | 18.50 | 22.58 | 19.42 | 16.27 |
| *Actinobacteriota* | 1.07 | 0.24 | 4.31 | 1.95 | 2.33 | 1.05 | 1.51 | 0.41 | 0.48 | 1.31 | 0.83 | 2.89 |
| *Acidobacteriota* | 0.11 | 0.07 | 3.65 | 2.35 | 0.03 | 0.59 | 0.30 | 0.01 | 0.79 | 0.48 | 1.59 | 2.17 |
| *Verrucomicrobiota* | 0.13 | 0.04 | 0.71 | 4.38 | 0.23 | 1.16 | 0.36 | 0.06 | 1.13 | 0.92 | 1.92 | 1.02 |
| *Fusobacteriota* | 0.34 | 0.38 | 0.80 | 0.57 | 2.63 | 0.14 | 1.50 | 0.08 | 0.18 | 1.10 | 0.34 | 0.64 |
| *Patescibacteria* | 0.10 | 0.02 | 0.12 | 0.32 | 0.21 | 0.75 | 1.31 | 0.05 | 0.53 | 1.01 | 0.59 | 0.59 |
| *Desulfobacterota* | 0.00 | 0.00 | 0.00 | 0.52 | 0.01 | 1.27 | 0.05 | 0.17 | 0.48 | 0.23 | 1.71 | 0.54 |
| unclassified *Archaea* | 0.01 | 0.00 | 0.02 | 1.10 | 0.12 | 0.12 | 0.12 | 0.15 | 0.16 | 0.71 | 0.49 | 0.21 |
| unclassified Bacteria | 0.02 | 0.01 | 0.65 | 0.34 | 0.02 | 0.45 | 0.07 | 0.01 | 0.20 | 0.19 | 0.44 | 0.51 |
| *Bdellovibrionota* | 0.01 | 0.02 | 0.64 | 0.16 | 0.01 | 0.30 | 0.29 | 0.01 | 0.16 | 0.54 | 0.26 | 0.37 |
| *Campylobacterota* | 0.01 | 0.00 | 0.02 | 0.08 | 0.08 | 0.32 | 0.41 | 0.01 | 0.41 | 0.53 | 0.41 | 0.20 |
| *Planctomycetota* | 0.01 | 0.01 | 0.35 | 0.65 | 0.00 | 0.23 | 0.03 | 0.01 | 0.21 | 0.06 | 0.45 | 0.41 |
| *Gemmatimonadota* | 0.01 | 0.01 | 0.53 | 0.32 | 0.01 | 0.17 | 0.04 | 0.01 | 0.10 | 0.09 | 0.26 | 0.41 |
| *Deferribacterota* | 0.00 | 0.00 | 0.00 | 0.41 | 0.00 | 0.23 | 0.00 | 0.08 | 0.34 | 0.01 | 0.47 | 0.12 |
| *Myxococcota* | 0.01 | 0.01 | 0.10 | 0.25 | 0.00 | 0.29 | 0.02 | 0.00 | 0.11 | 0.18 | 0.19 | 0.39 |
| *Nitrospirota* | 0.01 | 0.00 | 0.67 | 0.13 | 0.00 | 0.09 | 0.01 | 0.00 | 0.14 | 0.02 | 0.17 | 0.21 |
| *Chloroflexi* | 0.00 | 0.00 | 0.37 | 0.19 | 0.01 | 0.14 | 0.06 | 0.00 | 0.10 | 0.06 | 0.15 | 0.19 |
| Others | 0.07 | 0.02 | 0.51 | 0.35 | 0.39 | 0.40 | 0.40 | 0.04 | 0.46 | 0.78 | 0.49 | 0.74 |
| Unknown | 0.03 | 0.01 | 0.03 | 3.01 | 0.08 | 0.28 | 0.09 | 0.25 | 0.53 | 0.99 | 1.30 | 0.37 |

Table S3 Relative abundance of bacteria at the phylum level of raw milk from different regions and types (＞0.1%).

Note: G-SN, buffalo milk from Guangxi; X-MN, horse milk from Xingjiang; X-LT, camel milk from Xinjiang; S-LN, donkey milk from Shandong; XJ, Holstein cow milk from Xinjiang; GD, Holstein cow milk from Guangdong; ZB, Holstein cow milk from Zibo; YT, Holstein cow milk from Yantai; JN, Holstein cow milk from Jinan; WF, Holstein cow milk from Weifang; QD, Holstein cow milk from Qingdao; DY, Holstein cow milk from Dongying.
