# Supplementary material for: Mapping bacterial microbiota variations in raw milk: geographic and type-specific insights
Source: Microbiol Spectr. 2025 Oct 27;13(12):e00933-25. doi: 10.1128/spectrum.00933-25 (PMC12671074; doi:10.1128/spectrum.00933-25)
Supplement: Table S4 — Relative abundance of bacteria at the genus level of raw milk from different regions and types (>0.1). [file spectrum.00933-25-s0005.docx]

Table S4 Relative abundance of bacteria at the genus level of raw milk from different regions and types (＞0.1).

| genus | G-SN* (%) | X-MN* (%) | X-LT* (%) | S-LN* (%) | XJ* (%) | GD* (%) | ZB* (%) | YT* (%) | JN* (%) | WF* (%) | QD* (%) | DY* (%) |
| --- | --- | --- | --- | --- | --- | --- | --- | --- | --- | --- | --- | --- |
| *Acinetobacter* | 1.11E+01 | 6.98E+01 | 1.14E+01 | 1.19E+00 | 1.42E+00 | 7.76E-01 | 2.03E+01 | 2.97E+01 | 8.23E-01 | 9.46E+00 | 2.16E+00 | 4.97E+00 |
| *Pseudomonas* | 6.28E+01 | 6.27E-02 | 6.94E+00 | 3.53E+00 | 4.67E-01 | 8.28E-01 | 6.99E+00 | 4.48E+01 | 1.09E+00 | 7.40E+00 | 1.17E+00 | 1.17E+01 |
| *Lactiplantibacillus* | 4.96E-03 | 1.28E-03 | 1.01E-02 | 4.13E-01 | 3.43E-03 | 2.98E+01 | 1.00E-02 | 2.37E-02 | 3.89E+01 | 7.18E-01 | 2.27E+01 | 1.08E+01 |
| *Chryseobacterium* | 2.07E+00 | 4.74E-01 | 9.48E-01 | 6.55E-01 | 2.89E+01 | 5.62E-01 | 1.26E+01 | 4.45E+00 | 6.10E-01 | 1.21E+01 | 8.30E-01 | 6.59E+00 |
| *Enterobacter* | 9.88E-01 | 2.11E+01 | 6.45E+00 | 1.10E+00 | 3.18E-01 | 1.83E+00 | 3.36E-01 | 3.23E-01 | 1.92E+00 | 8.06E-01 | 1.56E+00 | 1.18E+00 |
| *Enhydrobacter* | 1.69E+00 | 1.71E+00 | 8.70E+00 | 1.13E-01 | 6.70E+00 | 1.29E-01 | 5.18E+00 | 2.04E-01 | 8.37E-02 | 6.01E+00 | 2.19E-01 | 4.66E+00 |
| *Streptococcus* | 3.54E+00 | 1.06E-01 | 1.32E+00 | 4.81E+00 | 1.06E+01 | 2.87E-01 | 1.05E+00 | 6.93E-01 | 1.02E+00 | 7.85E+00 | 1.21E+00 | 9.58E-01 |
| *Escherichia Shigella* | 2.98E-02 | 4.10E-02 | 6.48E-01 | 1.93E+00 | 1.35E+01 | 8.10E-01 | 8.40E-01 | 4.58E-01 | 5.72E-01 | 3.75E+00 | 2.14E+00 | 7.84E+00 |
| *Achromobacter* | 3.00E-01 | 2.93E-01 | 2.73E+00 | 1.17E+01 | 1.36E+00 | 2.49E+00 | 3.00E+00 | 2.37E-02 | 2.84E+00 | 1.74E+00 | 1.97E+00 | 1.09E+00 |
| *Lactobacillus* | 6.09E-01 | 4.29E-01 | 4.51E+00 | 7.69E+00 | 1.00E+00 | 4.29E-01 | 3.03E+00 | 4.99E-01 | 3.31E-01 | 2.80E+00 | 1.08E+00 | 3.00E+00 |
| unclassified *Muribaculaceae* | 2.73E-02 | 3.84E-02 | 9.28E-02 | 6.43E-01 | 1.84E-01 | 4.87E+00 | 1.38E-01 | 3.10E-01 | 5.23E+00 | 5.04E-01 | 5.21E+00 | 1.43E+00 |
| *Lactococcus* | 3.49E-01 | 1.24E-01 | 2.42E-01 | 3.03E-01 | 1.74E+00 | 4.67E-01 | 6.17E-01 | 3.52E+00 | 4.01E-01 | 3.22E+00 | 6.73E-01 | 2.65E+00 |
| *Leuconostoc* | 1.36E-02 | 6.27E-02 | 9.30E+00 | 4.45E-02 | 1.27E-01 | 5.53E-01 | 1.77E-02 | 5.04E-02 | 4.22E-01 | 8.58E-01 | 3.69E-01 | 1.97E-01 |
| *Bacteroides* | 1.36E-02 | 2.18E-02 | 2.17E-02 | 6.80E-01 | 8.61E-01 | 1.48E+00 | 7.45E-01 | 3.70E-01 | 1.50E+00 | 9.76E-01 | 2.01E+00 | 2.86E+00 |
| *Mycoplasma* | 2.05E-01 | 2.39E-01 | 4.31E-01 | 8.86E-01 | 1.21E-01 | 1.43E+00 | 4.54E-01 | 2.17E+00 | 1.85E+00 | 8.65E-01 | 1.74E+00 | 1.05E+00 |
| *Limosilactobacillus* | 3.91E-01 | 9.22E-02 | 3.56E+00 | 1.44E+00 | 1.03E-02 | 2.32E-01 | 1.90E+00 | 1.16E-01 | 2.44E-01 | 9.06E-01 | 3.39E-01 | 1.22E+00 |
| *Pantoea* | 7.04E-01 | 7.68E-03 | 7.29E+00 | 4.45E-02 | 0.00E+00 | 6.60E-01 | 1.54E-02 | 5.45E-01 | 5.41E-01 | 1.77E-02 | 4.21E-01 | 1.68E-01 |
| *Flavobacterium* | 4.60E-01 | 1.28E-03 | 5.80E-03 | 2.39E-01 | 9.39E-02 | 1.27E+00 | 1.93E+00 | 1.12E-01 | 7.92E-01 | 3.58E+00 | 5.99E-01 | 4.24E-01 |
| *Psychrobacter* | 2.48E-03 | 0.00E+00 | 2.23E+00 | 1.98E-01 | 2.34E+00 | 3.86E-02 | 2.38E+00 | 1.12E+00 | 6.98E-02 | 5.36E-01 | 1.66E-01 | 1.91E-01 |
| *Lentilactobacillus* | 1.66E-01 | 2.30E-02 | 1.17E+00 | 4.39E+00 | 6.87E-03 | 1.84E-01 | 7.58E-01 | 1.30E-01 | 3.87E-01 | 5.59E-01 | 2.14E-01 | 3.15E-01 |
| *Secundilactobacillus* | 4.96E-03 | 1.28E-03 | 2.61E-02 | 5.26E-02 | 0.00E+00 | 2.53E+00 | 1.00E-02 | 4.11E-03 | 2.72E+00 | 1.10E-01 | 2.09E+00 | 6.97E-01 |
| *Carnobacterium* | 4.96E-03 | 1.28E-03 | 2.90E-03 | 0.00E+00 | 1.26E-02 | 2.14E-02 | 7.56E+00 | 3.99E-01 | 0.00E+00 | 3.15E-02 | 5.88E-03 | 5.16E-02 |
| *Staphylococcus* | 2.35E+00 | 9.65E-01 | 1.19E+00 | 1.57E+00 | 2.99E-01 | 8.58E-02 | 2.48E-01 | 3.68E-01 | 7.32E-02 | 1.94E-01 | 2.00E-01 | 3.67E-01 |
| *Lachnospiraceae* NK4A136 group | 2.48E-03 | 6.40E-03 | 7.25E-03 | 1.58E-01 | 4.47E-02 | 1.80E+00 | 3.62E-02 | 2.21E-01 | 1.91E+00 | 4.33E-01 | 2.27E+00 | 4.93E-01 |
| genus | G-SN* (%) | X-MN* (%) | X-LT* (%) | S-LN* (%) | XJ* (%) | GD* (%) | ZB* (%) | YT* (%) | JN* (%) | WF* (%) | QD* (%) | DY* (%) |
| *Acetobacter* | 3.35E-02 | 3.46E-02 | 3.36E-01 | 5.89E+00 | 4.58E-03 | 0.00E+00 | 2.45E-01 | 3.95E-01 | 0.00E+00 | 2.61E-01 | 2.52E-03 | 8.82E-02 |
| *Levilactobacillus* | 2.36E-02 | 5.12E-03 | 1.25E-01 | 2.26E+00 | 0.00E+00 | 1.48E+00 | 7.94E-02 | 1.03E-02 | 1.52E+00 | 7.86E-02 | 1.23E+00 | 4.17E-01 |
| *Sphingobacterium* | 7.28E-01 | 1.54E-02 | 1.45E-03 | 4.45E-02 | 1.43E+00 | 1.16E+00 | 2.16E+00 | 4.53E-02 | 5.37E-01 | 3.53E-01 | 3.82E-01 | 1.65E-01 |
| unclassified *Lachnospiraceae* | 3.10E-02 | 1.28E-03 | 5.36E-02 | 1.74E-01 | 6.18E-01 | 1.44E+00 | 3.03E-01 | 2.96E-01 | 1.54E+00 | 4.15E-01 | 1.59E+00 | 5.16E-01 |
| uncultured *Bacteroidales bacterium* | 2.48E-03 | 7.68E-03 | 1.01E-02 | 4.09E-01 | 1.72E-02 | 1.68E+00 | 2.00E-02 | 6.07E-02 | 1.89E+00 | 1.57E-01 | 1.85E+00 | 4.81E-01 |
| *Fusobacterium* | 2.30E-01 | 2.24E-01 | 5.09E-01 | 4.73E-01 | 2.59E+00 | 8.58E-02 | 1.26E+00 | 4.83E-02 | 5.93E-02 | 2.90E-01 | 9.33E-02 | 3.46E-01 |
| *Rothia* | 1.50E-01 | 2.43E-02 | 3.77E+00 | 4.85E-02 | 2.52E-02 | 0.00E+00 | 4.62E-02 | 2.16E-02 | 0.00E+00 | 2.75E-02 | 3.87E-02 | 1.86E+00 |
| *Empedobacter* | 1.25E+00 | 0.00E+00 | 1.45E-03 | 0.00E+00 | 0.00E+00 | 9.05E-01 | 9.54E-01 | 2.37E-02 | 1.09E+00 | 2.49E-01 | 7.62E-01 | 3.14E-01 |
| *Akkermansia* | 1.12E-02 | 2.56E-03 | 3.48E-02 | 3.06E+00 | 8.59E-02 | 3.09E-01 | 2.24E-02 | 3.29E-02 | 2.58E-01 | 2.82E-01 | 7.75E-01 | 1.84E-01 |
| *Enterococcus* | 5.09E-02 | 1.84E+00 | 2.42E-01 | 1.98E-01 | 2.28E-01 | 7.72E-02 | 6.51E-01 | 8.54E-02 | 5.23E-02 | 8.50E-01 | 2.61E-01 | 4.41E-01 |
| *Serratia* | 1.61E-01 | 3.07E-02 | 9.08E-01 | 5.46E-01 | 5.04E-02 | 1.46E-01 | 3.24E-02 | 2.13E+00 | 1.29E-01 | 3.68E-01 | 2.02E-01 | 1.35E-01 |
| *Bacillus* | 8.67E-01 | 3.59E-02 | 6.55E-01 | 1.62E+00 | 7.10E-02 | 5.57E-02 | 2.71E-01 | 4.63E-02 | 4.19E-02 | 2.82E-01 | 2.56E-01 | 2.40E-01 |
| uncultured rumen bacterium | 8.31E-02 | 2.56E-03 | 6.09E-02 | 5.87E-01 | 6.13E-01 | 1.67E-01 | 4.76E-01 | 1.51E-01 | 4.36E-01 | 5.60E-01 | 7.63E-01 | 4.09E-01 |
| UCG 005 | 4.84E-02 | 5.12E-03 | 4.49E-02 | 1.90E-01 | 2.00E+00 | 2.57E-02 | 9.98E-01 | 1.44E-02 | 5.93E-02 | 5.60E-01 | 1.08E-01 | 2.22E-01 |
| *Clostridium* sensu stricto 1 | 1.12E-02 | 1.66E-02 | 9.86E-02 | 6.35E-01 | 3.87E-01 | 1.46E-01 | 9.56E-02 | 2.06E-02 | 2.41E-01 | 1.73E+00 | 3.38E-01 | 4.51E-01 |
| *Ligilactobacillus* | 4.84E-02 | 1.13E-01 | 1.23E-01 | 7.48E-01 | 1.14E-03 | 2.70E-01 | 1.18E-01 | 2.06E-02 | 3.98E-01 | 1.02E-01 | 2.01E+00 | 1.66E-01 |
| *Paucilactobacillus* | 3.72E-03 | 0.00E+00 | 5.80E-03 | 6.47E-02 | 1.14E-03 | 1.13E+00 | 5.40E-03 | 1.23E-02 | 1.18E+00 | 6.78E-02 | 1.05E+00 | 3.41E-01 |
| *Macrococcus* | 8.44E-02 | 2.43E-02 | 1.85E+00 | 2.02E-02 | 4.24E-02 | 8.58E-03 | 5.01E-02 | 6.07E-02 | 0.00E+00 | 1.89E-01 | 4.37E-02 | 1.44E+00 |
| *Alistipes* | 2.85E-02 | 1.28E-03 | 1.01E-02 | 3.56E-01 | 2.04E-01 | 7.46E-01 | 1.36E-01 | 3.81E-02 | 7.78E-01 | 2.00E-01 | 9.40E-01 | 2.35E-01 |
| *Alloprevotella* | 1.24E-03 | 1.41E-02 | 5.07E-02 | 6.07E-02 | 5.04E-02 | 9.26E-01 | 1.46E-02 | 6.38E-02 | 1.09E+00 | 9.44E-02 | 9.59E-01 | 2.89E-01 |
| *Kluyvera* | 1.27E+00 | 1.20E-01 | 1.37E+00 | 1.62E-02 | 0.00E+00 | 1.07E-01 | 8.48E-03 | 4.01E-02 | 1.71E-01 | 2.25E-01 | 1.45E-01 | 3.33E-02 |
| *Lacticaseibacillus* | 0.00E+00 | 0.00E+00 | 1.45E-02 | 4.45E-02 | 1.26E-02 | 1.13E+00 | 3.47E-02 | 0.00E+00 | 1.01E+00 | 5.31E-02 | 7.97E-01 | 2.19E-01 |
| *Rikenellaceae* RC9 gut group | 3.23E-02 | 1.28E-03 | 4.64E-02 | 1.42E-01 | 1.32E+00 | 3.00E-02 | 6.34E-01 | 3.81E-02 | 1.26E-01 | 3.92E-01 | 1.86E-01 | 2.66E-01 |
| *Sphingomonas* | 1.24E-02 | 1.41E-02 | 7.13E-01 | 5.70E-01 | 1.14E-02 | 3.56E-01 | 7.09E-02 | 5.66E-02 | 3.59E-01 | 1.06E-01 | 4.02E-01 | 5.39E-01 |
| unclassified *Archaea* | 1.49E-02 | 0.00E+00 | 1.88E-02 | 1.10E+00 | 1.19E-01 | 1.16E-01 | 1.23E-01 | 1.47E-01 | 1.60E-01 | 7.10E-01 | 4.86E-01 | 2.09E-01 |
| genus | G-SN* (%) | X-MN* (%) | X-LT* (%) | S-LN* (%) | XJ* (%) | GD* (%) | ZB* (%) | YT* (%) | JN* (%) | WF* (%) | QD* (%) | DY* (%) |
| *Stenotrophomonas* | 1.58E-01 | 6.40E-03 | 5.80E-03 | 6.47E-02 | 3.66E-02 | 7.08E-01 | 2.36E-01 | 1.12E-01 | 6.77E-01 | 1.89E-01 | 6.14E-01 | 3.66E-01 |
| *Bradyrhizobium* | 4.84E-02 | 1.02E-02 | 3.48E-02 | 1.53E+00 | 4.58E-03 | 1.24E-01 | 3.40E-01 | 1.54E-02 | 1.19E-01 | 7.18E-01 | 1.24E-01 | 9.20E-02 |
| unclassified Bacteria | 1.74E-02 | 8.96E-03 | 6.45E-01 | 3.40E-01 | 2.06E-02 | 4.50E-01 | 6.71E-02 | 7.20E-03 | 1.99E-01 | 1.94E-01 | 4.43E-01 | 5.13E-01 |
| *Paucibacter* | 0.00E+00 | 0.00E+00 | 5.36E-02 | 1.90E-01 | 5.72E-03 | 2.87E-01 | 3.85E-03 | 4.83E-02 | 1.09E+00 | 3.21E-01 | 5.49E-01 | 3.48E-01 |
| unclassified *Vicinamibacteraceae* | 1.12E-02 | 8.96E-03 | 7.74E-01 | 1.90E-01 | 4.58E-03 | 1.72E-01 | 2.00E-02 | 0.00E+00 | 1.85E-01 | 4.62E-02 | 4.31E-01 | 5.95E-01 |
| *Odoribacter* | 0.00E+00 | 0.00E+00 | 0.00E+00 | 5.26E-02 | 0.00E+00 | 5.40E-01 | 8.48E-03 | 2.06E-03 | 7.60E-01 | 3.54E-02 | 8.14E-01 | 1.38E-01 |
| *Atopostipes* | 0.00E+00 | 7.68E-03 | 1.54E-01 | 2.43E-02 | 1.71E+00 | 0.00E+00 | 2.53E-01 | 1.03E-03 | 0.00E+00 | 6.39E-02 | 8.41E-03 | 4.27E-02 |
| *Porphyromonas* | 0.00E+00 | 1.28E-03 | 5.36E-02 | 3.32E-01 | 9.07E-01 | 8.58E-03 | 8.76E-01 | 6.17E-03 | 0.00E+00 | 1.18E-02 | 1.68E-02 | 1.83E-02 |
| *Desulfovibrio* | 0.00E+00 | 0.00E+00 | 0.00E+00 | 3.11E-01 | 9.16E-03 | 2.23E-01 | 1.00E-02 | 1.85E-02 | 2.27E-01 | 4.82E-02 | 1.23E+00 | 1.45E-01 |
| unclassified *Vicinamibacterales* | 2.61E-02 | 1.28E-02 | 4.87E-01 | 6.11E-01 | 0.00E+00 | 1.29E-01 | 4.78E-02 | 0.00E+00 | 1.08E-01 | 1.25E-01 | 2.44E-01 | 3.96E-01 |
| *Prevotellaceae* UCG 001 | 1.24E-03 | 0.00E+00 | 8.70E-03 | 6.47E-02 | 8.47E-02 | 4.80E-01 | 2.62E-02 | 1.85E-02 | 5.69E-01 | 7.37E-02 | 5.81E-01 | 1.27E-01 |
| *Furfurilactobacillus* | 0.00E+00 | 0.00E+00 | 0.00E+00 | 8.09E-03 | 0.00E+00 | 6.60E-01 | 0.00E+00 | 0.00E+00 | 6.45E-01 | 3.44E-02 | 4.84E-01 | 1.33E-01 |
| *Pedobacter* | 2.11E-02 | 1.28E-03 | 5.36E-02 | 1.17E-01 | 2.18E-02 | 7.80E-01 | 1.80E-01 | 0.00E+00 | 2.48E-01 | 1.66E-01 | 1.75E-01 | 9.86E-02 |
| RB41 | 2.48E-03 | 1.54E-02 | 8.15E-01 | 2.63E-01 | 6.87E-03 | 4.29E-02 | 3.08E-02 | 3.09E-03 | 8.37E-02 | 3.15E-02 | 1.87E-01 | 3.69E-01 |
| TM7a | 1.24E-03 | 1.28E-03 | 5.80E-03 | 2.83E-02 | 8.13E-02 | 1.72E-01 | 6.19E-01 | 8.23E-03 | 2.27E-01 | 4.35E-01 | 1.17E-01 | 7.74E-02 |
| *Christensenellaceae* R 7 group | 1.12E-02 | 1.28E-03 | 4.64E-02 | 1.50E-01 | 4.17E-01 | 1.72E-02 | 3.63E-01 | 1.03E-03 | 7.32E-02 | 3.23E-01 | 1.35E-01 | 1.86E-01 |
| *Mucispirillum* | 0.00E+00 | 0.00E+00 | 0.00E+00 | 4.13E-01 | 0.00E+00 | 2.32E-01 | 0.00E+00 | 8.02E-02 | 3.42E-01 | 1.18E-02 | 4.72E-01 | 1.21E-01 |
| *Sediminibacterium* | 0.00E+00 | 0.00E+00 | 0.00E+00 | 4.05E-03 | 0.00E+00 | 5.49E-01 | 0.00E+00 | 0.00E+00 | 5.23E-01 | 9.83E-03 | 4.75E-01 | 1.01E-01 |
| *Microbacterium* | 2.52E-01 | 2.43E-02 | 1.16E-02 | 3.64E-02 | 8.82E-02 | 1.54E-01 | 6.15E-01 | 1.09E-01 | 5.23E-02 | 9.63E-02 | 8.74E-02 | 1.30E-01 |
| *Pasteurella* | 0.00E+00 | 0.00E+00 | 1.74E-02 | 8.09E-03 | 1.54E+00 | 0.00E+00 | 0.00E+00 | 0.00E+00 | 0.00E+00 | 6.98E-02 | 4.20E-03 | 7.51E-03 |
| *Cetobacterium* | 1.07E-01 | 1.45E-01 | 2.64E-01 | 1.21E-02 | 0.00E+00 | 1.29E-02 | 2.44E-01 | 2.26E-02 | 8.02E-02 | 2.31E-01 | 2.35E-01 | 2.76E-01 |
| *Halomonas* | 1.24E-02 | 2.56E-03 | 8.70E-02 | 2.02E-01 | 1.79E-01 | 1.11E-01 | 2.56E-01 | 9.26E-03 | 1.05E-01 | 3.64E-01 | 6.98E-02 | 2.14E-01 |
| unclassified *Gemmatimonadaceae* | 8.69E-03 | 8.96E-03 | 4.74E-01 | 2.63E-01 | 4.58E-03 | 1.37E-01 | 2.08E-02 | 1.03E-02 | 9.77E-02 | 3.44E-02 | 2.18E-01 | 3.26E-01 |
| *Trueperella* | 3.72E-03 | 0.00E+00 | 0.00E+00 | 4.05E-03 | 1.50E+00 | 0.00E+00 | 4.62E-02 | 0.00E+00 | 0.00E+00 | 3.64E-02 | 0.00E+00 | 4.69E-04 |
| *Rahnella1* | 7.99E-01 | 0.00E+00 | 2.90E-03 | 0.00E+00 | 2.29E-03 | 0.00E+00 | 3.62E-02 | 2.56E-01 | 0.00E+00 | 2.46E-01 | 1.01E-02 | 1.99E-01 |
| genus | G-SN* (%) | X-MN* (%) | X-LT* (%) | S-LN* (%) | XJ* (%) | GD* (%) | ZB* (%) | YT* (%) | JN* (%) | WF* (%) | QD* (%) | DY* (%) |
| unclassified *Chitinophagaceae* | 1.99E-02 | 1.15E-02 | 3.33E-01 | 3.72E-01 | 3.43E-03 | 9.43E-02 | 3.70E-02 | 2.06E-03 | 1.22E-01 | 1.49E-01 | 1.76E-01 | 2.29E-01 |
| unclassified [*Eubacterium*] coprostanoligenes group | 9.93E-03 | 0.00E+00 | 2.46E-02 | 1.58E-01 | 3.18E-01 | 2.06E-01 | 1.64E-01 | 1.11E-01 | 1.19E-01 | 1.53E-01 | 1.53E-01 | 1.17E-01 |
| unclassified *Desulfovibrionaceae* | 0.00E+00 | 0.00E+00 | 1.45E-03 | 1.66E-01 | 0.00E+00 | 3.26E-01 | 0.00E+00 | 3.50E-02 | 2.41E-01 | 8.26E-02 | 3.59E-01 | 3.03E-01 |
| *Nitrospira* | 9.93E-03 | 2.56E-03 | 6.73E-01 | 1.29E-01 | 4.58E-03 | 7.72E-02 | 1.16E-02 | 1.03E-03 | 1.43E-01 | 1.67E-02 | 1.72E-01 | 2.07E-01 |
| *Terrimonas* | 1.36E-02 | 1.28E-02 | 2.31E-01 | 3.40E-01 | 5.72E-03 | 1.37E-01 | 7.32E-02 | 2.06E-03 | 1.64E-01 | 1.85E-01 | 1.73E-01 | 8.31E-02 |
| *Delftia* | 3.72E-02 | 1.54E-02 | 1.39E-01 | 4.61E-01 | 8.24E-02 | 3.43E-02 | 3.31E-01 | 5.97E-02 | 3.49E-02 | 1.09E-01 | 2.94E-02 | 5.54E-02 |
| *Companilactobacillus* | 0.00E+00 | 1.28E-03 | 0.00E+00 | 7.28E-02 | 0.00E+00 | 4.42E-01 | 6.94E-03 | 0.00E+00 | 4.22E-01 | 1.77E-02 | 3.19E-01 | 8.50E-02 |
| unclassified *Clostridia* UCG 014 | 6.20E-03 | 2.30E-02 | 2.61E-02 | 1.58E-01 | 1.03E-01 | 1.67E-01 | 6.86E-02 | 1.31E-01 | 1.88E-01 | 1.18E-01 | 3.21E-01 | 5.40E-02 |
| *Blautia* | 1.24E-03 | 3.84E-03 | 8.70E-03 | 1.17E-01 | 5.04E-02 | 3.09E-01 | 1.39E-02 | 1.15E-01 | 2.96E-01 | 6.68E-02 | 2.84E-01 | 9.57E-02 |
| unclassified *Xanthobacteraceae* | 5.34E-02 | 7.68E-03 | 3.91E-02 | 9.06E-01 | 0.00E+00 | 3.43E-02 | 3.08E-02 | 4.11E-03 | 2.09E-02 | 1.36E-01 | 5.55E-02 | 4.36E-02 |
| *Comamonas* | 1.81E-01 | 2.50E-01 | 5.80E-03 | 7.69E-02 | 3.13E-01 | 4.29E-03 | 1.16E-01 | 9.26E-02 | 6.98E-03 | 1.76E-01 | 7.56E-03 | 7.51E-02 |
| *Prevotella* | 2.98E-02 | 1.79E-02 | 3.48E-02 | 6.07E-02 | 8.01E-02 | 3.00E-02 | 4.55E-02 | 2.78E-02 | 2.41E-01 | 2.77E-01 | 2.71E-01 | 1.72E-01 |
| *Helicobacter* | 0.00E+00 | 0.00E+00 | 4.35E-03 | 6.07E-02 | 0.00E+00 | 2.87E-01 | 7.71E-04 | 6.17E-03 | 3.87E-01 | 4.23E-02 | 3.95E-01 | 7.65E-02 |
| *Massilia* | 3.47E-02 | 1.28E-03 | 9.71E-02 | 2.31E-01 | 7.10E-02 | 4.72E-02 | 9.64E-02 | 2.48E-01 | 1.12E-01 | 6.29E-02 | 1.17E-01 | 1.40E-01 |
| *Ulvibacter* | 0.00E+00 | 2.56E-03 | 0.00E+00 | 4.45E-02 | 1.72E-02 | 1.14E+00 | 0.00E+00 | 0.00E+00 | 1.05E-02 | 0.00E+00 | 3.36E-02 | 0.00E+00 |
| *Parabacteroides* | 4.96E-03 | 0.00E+00 | 2.90E-03 | 8.50E-02 | 1.26E-02 | 2.66E-01 | 5.40E-03 | 3.39E-02 | 2.37E-01 | 7.37E-02 | 3.70E-01 | 1.61E-01 |
| unclassified UCG 010 | 2.23E-02 | 0.00E+00 | 2.61E-02 | 5.66E-02 | 3.45E-01 | 3.86E-02 | 3.01E-01 | 1.34E-02 | 2.79E-02 | 1.92E-01 | 5.72E-02 | 1.24E-01 |
| Others | 5.11E+00 | 1.26E+00 | 1.41E+01 | 2.53E+01 | 1.19E+01 | 2.40E+01 | 1.53E+01 | 3.62E+00 | 1.28E+01 | 1.92E+01 | 2.07E+01 | 1.77E+01 |
| Unknown | 3.23E-02 | 7.68E-03 | 3.04E-02 | 3.01E+00 | 7.67E-02 | 2.79E-01 | 8.94E-02 | 2.49E-01 | 5.27E-01 | 9.88E-01 | 1.30E+00 | 3.72E-01 |

Note: G-SN, buffalo milk from Guangxi; X-MN, horse milk from Xingjiang; X-LT, camel milk from Xinjiang; S-LN, donkey milk from Shandong; XJ, Holstein cow milk from Xinjiang; GD, Holstein cow milk from GuangDong; ZB, Holstein cow milk from Zibo; YT, Holstein cow milk from Yantai; JN*, Holstein cow milk from Jinan; WF, Holstein cow milk from Weifang; QD, Holstein cow milk from QingDao; DY, Holstein cow milk from Dongying.
