# Supplementary material for: Mapping bacterial microbiota variations in raw milk: geographic and type-specific insights
Source: Microbiol Spectr. 2025 Oct 27;13(12):e00933-25. doi: 10.1128/spectrum.00933-25 (PMC12671074; doi:10.1128/spectrum.00933-25)
Supplement: Table S5 — Relative abundance of bacteria at the species level of raw milk from different regions and types (>0.1%). [file spectrum.00933-25-s0006.docx]

Table S5 Relative abundance of bacteria at the species level of raw milk from different regions and types (＞0.1%).

| species | G-SN* (%) | X-MN* (%) | X-LT* (%) | S-LN* (%) | XJ* (%) | GD* (%) | ZB* (%) | YT* (%) | JN* (%) | WF* (%) | QD* (%) | DY* (%) |
| --- | --- | --- | --- | --- | --- | --- | --- | --- | --- | --- | --- | --- |
| *Lactiplantibacillus plantarum* | 4.96E-03 | 1.28E-03 | 1.01E-02 | 4.13E-01 | 3.43E-03 | 2.98E+01 | 1.00E-02 | 2.37E-02 | 3.89E+01 | 7.18E-01 | 2.27E+01 | 1.08E+01 |
| *Acinetobacter johnsonii* | 8.22 | 6.95E+01 | 9.41 | 1.66E-01 | 8.05E-01 | 4.33E-01 | 1.76 | 5.72E-01 | 5.30E-01 | 9.51E-01 | 4.42E-01 | 9.42E-01 |
| *Pseudomonas fragi* | 5.43E+01 | 0 | 5.48 | 1.42 | 1.75E-01 | 4.72E-02 | 5.63E-02 | 1.18E+01 | 1.81E-01 | 3.70E-01 | 2.60E-01 | 6.47 |
| *Pseudomonas lurida* | 4.76 | 5.38E-02 | 2.77E-01 | 1.20 | 1.68E-01 | 2.27E-01 | 6.66 | 3.29E+01 | 4.19E-01 | 6.32 | 4.85E-01 | 4.81 |
| *Acinetobacter albensis* | 1.24E-03 | 1.28E-03 | 2.90E-03 | 0 | 5.27E-02 | 2.14E-02 | 1.71E+01 | 2.75E+01 | 1.05E-02 | 6.90 | 1.27 | 8.87E-01 |
| *Enterobacter ludwigii* | 9.88E-01 | 2.11E+01 | 6.45 | 1.10 | 3.18E-01 | 1.83 | 3.36E-01 | 3.23E-01 | 1.92 | 8.06E-01 | 1.56 | 1.18 |
| *Moraxella osloensis* | 1.69 | 1.71 | 8.70 | 8.09E-02 | 6.70 | 1.20E-01 | 5.18 | 2.00E-01 | 2.44E-02 | 6.01 | 2.15E-01 | 4.65 |
| *Escherichia coli* | 2.98E-02 | 4.10E-02 | 6.48E-01 | 1.93 | 1.35E+01 | 8.10E-01 | 8.40E-01 | 4.53E-01 | 5.72E-01 | 3.75 | 2.14 | 7.84 |
| *Achromobacter arsenitoxydans* | 3.00E-01 | 2.93E-01 | 2.73 | 1.17E+01 | 1.36 | 2.49 | 3.00 | 2.37E-02 | 2.84 | 1.74 | 1.97 | 1.09 |
| *Chryseobacterium carnipullorum* | 5.96E-01 | 0 | 4.35E-03 | 0 | 2.49E+01 | 0 | 3.30 | 1.03E-02 | 0 | 2.89E-01 | 8.41E-03 | 1.50E-02 |
| unclassified *Muribaculaceae* | 2.73E-02 | 3.84E-02 | 9.28E-02 | 6.43E-01 | 1.84E-01 | 4.87 | 1.38E-01 | 3.10E-01 | 5.23 | 5.04E-01 | 5.21 | 1.43 |
| *Haloanella gallinarum* | 9.19E-01 | 3.84E-03 | 5.94E-01 | 3.24E-02 | 2.29 | 0 | 1.66 | 3.89E-01 | 0 | 8.91 | 7.48E-02 | 2.22 |
| *Lactobacillus helveticus* | 4.38E-01 | 4.14E-01 | 2.89 | 5.55 | 7.68E-01 | 5.15E-02 | 1.98 | 2.85E-01 | 1.26E-01 | 1.49 | 1.14E-01 | 7.92E-01 |
| uncultured *Bacteroidales bacterium* | 3.35E-02 | 7.68E-03 | 2.32E-02 | 4.65E-01 | 1.47 | 2.61 | 6.76E-01 | 1.24E-01 | 2.98 | 6.07E-01 | 2.78 | 8.57E-01 |
| *Leuconostoc mesenteroides* | 1.36E-02 | 6.27E-02 | 9.30 | 4.45E-02 | 1.27E-01 | 5.53E-01 | 1.77E-02 | 5.04E-02 | 4.22E-01 | 8.58E-01 | 3.69E-01 | 1.97E-01 |
| *Mycoplasma wenyonii* | 2.05E-01 | 2.36E-01 | 4.25E-01 | 8.50E-01 | 1.10E-01 | 1.39 | 4.45E-01 | 2.17 | 1.83 | 8.36E-01 | 1.65 | 1.02 |
| *Limosilactobacillus pontis* | 3.28E-01 | 1.15E-02 | 3.25 | 1.08 | 0 | 9.01E-02 | 1.69 | 2.37E-02 | 2.09E-02 | 7.21E-01 | 5.30E-02 | 1.09 |
| *Secundilactobacillus odoratitofui* | 4.96E-03 | 1.28E-03 | 2.61E-02 | 5.26E-02 | 0 | 2.53 | 1.00E-02 | 4.11E-03 | 2.72 | 1.10E-01 | 2.09 | 6.97E-01 |
| *Carnobacterium maltaromaticum* | 4.96E-03 | 0 | 2.90E-03 | 0 | 1.03E-02 | 1.72E-02 | 7.56 | 3.99E-01 | 0 | 3.15E-02 | 5.88E-03 | 5.16E-02 |
| *Streptococcaceae bacterium* | 3.10E-02 | 7.04E-02 | 6.38E-02 | 4.09E-01 | 0 | 7.29E-02 | 7.25E-02 | 6.99E-02 | 1.78E-01 | 6.29 | 4.98E-01 | 4.98E-02 |
| *Levilactobacillus brevis* | 2.36E-02 | 5.12E-03 | 1.25E-01 | 2.25 | 0 | 1.35 | 7.86E-02 | 1.03E-02 | 1.34 | 6.59E-02 | 1.07 | 3.68E-01 |
| *Acetobacter pasteurianus* | 3.10E-02 | 2.82E-02 | 3.20E-01 | 5.30 | 4.58E-03 | 0 | 2.40E-01 | 3.91E-01 | 0 | 2.16E-01 | 8.41E-04 | 7.32E-02 |
| *Pseudomonas laurentiana* | 3.71 | 5.12E-03 | 8.97E-01 | 6.63E-01 | 1.03E-02 | 1.11E-01 | 3.78E-02 | 1.70E-01 | 3.73E-01 | 5.80E-02 | 2.97E-01 | 2.27E-01 |
| unclassified *Lachnospiraceae* | 2.85E-02 | 1.28E-03 | 5.22E-02 | 1.50E-01 | 5.62E-01 | 1.28 | 2.82E-01 | 2.80E-01 | 1.44 | 3.58E-01 | 1.43 | 4.79E-01 |
| *Lactococcus piscium* | 0 | 0 | 0 | 0 | 3.38E-01 | 1.29E-02 | 2.47E-02 | 2.68 | 0 | 2.28 | 2.41E-01 | 7.06E-01 |
| species | G-SN* (%) | X-MN* (%) | X-LT* (%) | S-LN* (%) | XJ* (%) | GD* (%) | ZB* (%) | YT* (%) | JN* (%) | WF* (%) | QD* (%) | DY* (%) |
| *Pantoea ananatis* | 6.66E-01 | 7.68E-03 | 4.77 | 0 | 0 | 2.23E-01 | 3.08E-03 | 3.09E-03 | 1.67E-01 | 5.90E-03 | 1.83E-01 | 7.09E-02 |
| *Epilithonimonas bovis* | 6.20E-02 | 3.67E-01 | 0 | 4.85E-02 | 4.27E-01 | 5.15E-02 | 9.46E-01 | 1.34E-01 | 2.79E-02 | 7.46E-01 | 1.57E-01 | 3.06 |
| uncultured *rumen bacterium* | 1.01E-01 | 1.15E-02 | 9.28E-02 | 7.12E-01 | 8.32E-01 | 2.40E-01 | 5.48E-01 | 1.61E-01 | 6.98E-01 | 6.71E-01 | 1.08 | 7.12E-01 |
| *Rothia endophytica* | 1.27E-01 | 1.54E-02 | 3.69 | 2.83E-02 | 2.29E-02 | 0 | 4.62E-02 | 1.34E-02 | 0 | 2.16E-02 | 3.53E-02 | 1.85 |
| *Lactobacillus acetotolerans* | 1.64E-01 | 7.68E-03 | 1.61 | 1.54 | 2.29E-03 | 8.58E-03 | 9.47E-01 | 9.36E-02 | 0 | 6.82E-01 | 5.88E-03 | 4.31E-01 |
| *Staphylococcus epidermidis* | 1.28 | 8.03E-01 | 1.08 | 1.10 | 2.20E-01 | 8.58E-02 | 1.15E-01 | 9.26E-02 | 6.63E-02 | 1.15E-01 | 1.74E-01 | 3.22E-01 |
| *Acinetobacter guillouiae* | 1.68 | 2.32E-01 | 0 | 8.33E-01 | 1.83E-02 | 1.29E-02 | 2.86E-01 | 4.25E-01 | 6.98E-03 | 3.18E-01 | 4.45E-02 | 1.43 |
| *Lactococcus lactis* | 2.06E-01 | 6.79E-02 | 1.35E-01 | 2.51E-01 | 1.03 | 4.42E-01 | 3.54E-01 | 1.84E-01 | 3.98E-01 | 4.69E-01 | 3.61E-01 | 1.36 |
| *Psychrobacter alimentarius* | 0 | 0 | 1.08 | 1.01E-01 | 9.64E-01 | 2.14E-02 | 1.27 | 1.12 | 1.74E-02 | 4.61E-01 | 2.86E-02 | 1.15E-01 |
| *Streptococcus agalactiae* | 0 | 0 | 0 | 4.05E-03 | 4.65 | 0 | 0 | 2.06E-03 | 1.26E-01 | 0 | 8.74E-02 | 3.19E-02 |
| *Akkermansia muciniphila* | 1.24E-03 | 2.56E-03 | 1.30E-02 | 3.02 | 2.29E-03 | 3.09E-01 | 1.54E-03 | 2.78E-02 | 2.55E-01 | 2.48E-01 | 7.67E-01 | 1.61E-01 |
| *Streptococcus equinus* | 3.43 | 0 | 6.52E-02 | 0 | 1.66E-01 | 0 | 3.55E-02 | 3.34E-01 | 0 | 2.14E-01 | 5.04E-03 | 3.97E-01 |
| unclassified *Lachnospiraceae* NK4A136 group | 1.24E-03 | 5.12E-03 | 4.35E-03 | 9.30E-02 | 2.52E-02 | 1.10 | 2.24E-02 | 1.44E-01 | 1.11 | 2.82E-01 | 1.44 | 3.14E-01 |
| *Empedobacter stercoris* | 1.10 | 0 | 0 | 0 | 0 | 8.66E-01 | 2.14E-01 | 1.34E-02 | 1.05 | 1.83E-01 | 7.46E-01 | 2.63E-01 |
| uncultured *Ruminococcaceae bacterium* | 5.58E-02 | 8.96E-03 | 4.20E-02 | 7.28E-02 | 2.10 | 2.57E-02 | 1.15 | 1.03E-03 | 2.79E-02 | 5.51E-01 | 3.11E-02 | 2.91E-01 |
| endosymbiont of *Nilaparvata lugens* | 2.98E-02 | 0 | 1.45E-03 | 4.05E-03 | 7.89E-01 | 1.72E-02 | 2.58 | 1.95E-02 | 1.05E-02 | 5.80E-01 | 1.60E-02 | 3.06E-01 |
| *Lentilactobacillus buchneri* | 5.34E-02 | 1.15E-02 | 3.91E-01 | 2.51 | 6.87E-03 | 1.59E-01 | 2.38E-01 | 8.95E-02 | 3.59E-01 | 1.90E-01 | 1.91E-01 | 1.36E-01 |
| *Acinetobacter baumannii* | 3.28E-01 | 1.28E-03 | 6.44E-01 | 4.85E-02 | 3.06E-01 | 2.79E-01 | 1.26E-01 | 8.76E-01 | 2.69E-01 | 3.49E-01 | 3.21E-01 | 7.20E-01 |
| *Psychrobacter sanguinis* | 2.48E-03 | 0 | 1.16 | 9.71E-02 | 1.37 | 1.72E-02 | 1.11 | 0 | 2.09E-02 | 7.47E-02 | 5.80E-02 | 7.37E-02 |
| *Lentilactobacillus hilgardii* | 1.13E-01 | 1.15E-02 | 7.74E-01 | 1.85 | 0 | 0 | 5.20E-01 | 4.01E-02 | 0 | 3.69E-01 | 8.41E-04 | 1.74E-01 |
| *Lactobacillus vaccinostercus* DSM 20634 | 3.72E-03 | 0 | 5.80E-03 | 6.47E-02 | 1.14E-03 | 1.13 | 5.40E-03 | 3.09E-03 | 1.18 | 6.78E-02 | 1.05 | 3.30E-01 |
| *Macrococcus caseolyticus* | 8.44E-02 | 2.43E-02 | 1.85 | 2.02E-02 | 4.24E-02 | 8.58E-03 | 5.01E-02 | 6.07E-02 | 0 | 1.89E-01 | 4.37E-02 | 1.44 |
| *Streptococcus uberis* | 2.48E-02 | 0 | 1.17E-01 | 4.05E-03 | 2.72 | 0 | 8.15E-01 | 7.20E-03 | 1.05E-02 | 2.56E-02 | 3.36E-03 | 8.45E-03 |
| *Ligilactobacillus murinus* | 4.72E-02 | 9.35E-02 | 1.10E-01 | 3.80E-01 | 1.14E-03 | 2.70E-01 | 1.13E-01 | 1.85E-02 | 3.98E-01 | 9.63E-02 | 2.00 | 1.59E-01 |
| *Fusobacterium necrophorum* | 0 | 0 | 1.74E-02 | 1.38E-01 | 2.58 | 0 | 7.82E-01 | 2.06E-03 | 6.98E-03 | 0 | 8.41E-04 | 0 |
| species | G-SN* (%) | X-MN* (%) | X-LT* (%) | S-LN* (%) | XJ* (%) | GD* (%) | ZB* (%) | YT* (%) | JN* (%) | WF* (%) | QD* (%) | DY* (%) |
| *Chryseobacterium soli* | 0 | 0 | 0 | 0 | 0 | 0 | 4.39E-02 | 3.48 | 0 | 0 | 0 | 3.29E-03 |
| *Kluyvera ascorbata* | 1.27 | 1.20E-01 | 1.37 | 1.62E-02 | 0 | 1.07E-01 | 8.48E-03 | 4.01E-02 | 1.71E-01 | 2.25E-01 | 1.45E-01 | 3.33E-02 |
| *Sphingobacterium kitahiroshimense* | 2.17E-01 | 8.96E-03 | 1.45E-03 | 4.05E-02 | 1.41 | 0 | 1.75 | 3.29E-02 | 0 | 8.85E-03 | 1.68E-03 | 1.60E-02 |
| *Flavobacterium collinsii* | 0 | 1.28E-03 | 0 | 0 | 0 | 0 | 6.61E-01 | 0 | 1.05E-02 | 2.71 | 5.88E-03 | 9.39E-04 |
| unclassified *Archaea* | 1.49E-02 | 0 | 1.88E-02 | 1.10 | 1.19E-01 | 1.16E-01 | 1.23E-01 | 1.47E-01 | 1.60E-01 | 7.10E-01 | 4.86E-01 | 2.09E-01 |
| *Bradyrhizobium jicamae* | 4.84E-02 | 1.02E-02 | 3.48E-02 | 1.47 | 4.58E-03 | 1.24E-01 | 3.36E-01 | 1.54E-02 | 1.19E-01 | 6.90E-01 | 1.24E-01 | 9.20E-02 |
| *Lactobacillus johnsonii* | 4.96E-03 | 2.56E-03 | 7.25E-03 | 2.87E-01 | 1.26E-02 | 1.29E-01 | 1.54E-02 | 2.47E-02 | 9.07E-02 | 1.32E-01 | 6.26E-01 | 1.71 |
| *Serratia liquefaciens* | 6.70E-02 | 2.43E-02 | 5.89E-01 | 6.47E-02 | 6.87E-03 | 4.29E-03 | 1.46E-02 | 1.91 | 3.49E-03 | 2.60E-01 | 7.73E-02 | 1.36E-02 |
| *Bacteroides acidifaciens* | 1.24E-03 | 0 | 2.90E-03 | 2.22E-01 | 0 | 6.78E-01 | 0 | 1.07E-01 | 7.26E-01 | 3.34E-02 | 1.04 | 1.92E-01 |
| unclassified Bacteria | 1.74E-02 | 8.96E-03 | 6.45E-01 | 3.40E-01 | 2.06E-02 | 4.50E-01 | 6.71E-02 | 7.20E-03 | 1.99E-01 | 1.94E-01 | 4.43E-01 | 5.13E-01 |
| *Cenchrus americanus* | 0 | 0 | 5.36E-02 | 1.90E-01 | 5.72E-03 | 2.87E-01 | 3.85E-03 | 4.83E-02 | 1.09 | 2.97E-01 | 5.48E-01 | 3.37E-01 |
| *Clostridium disporicum* | 1.24E-03 | 0 | 9.71E-02 | 4.00E-01 | 2.66E-01 | 1.11E-01 | 6.47E-02 | 8.23E-03 | 1.88E-01 | 1.42 | 2.14E-01 | 8.87E-02 |
| unclassified *Vicinamibacterales* | 2.98E-02 | 1.54E-02 | 7.71E-01 | 6.92E-01 | 0 | 1.37E-01 | 6.24E-02 | 0 | 1.53E-01 | 1.57E-01 | 2.87E-01 | 5.04E-01 |
| *Stenotrophomonas maltophilia* | 7.07E-02 | 6.40E-03 | 1.45E-03 | 3.64E-02 | 2.98E-02 | 7.03E-01 | 1.34E-01 | 6.79E-02 | 6.77E-01 | 1.25E-01 | 5.77E-01 | 2.86E-01 |
| *Acinetobacter ursingii* | 2.88E-01 | 6.40E-03 | 1.02 | 4.05E-03 | 2.06E-02 | 1.72E-02 | 4.15E-01 | 8.85E-02 | 0 | 2.88E-01 | 2.44E-02 | 4.84E-01 |
| *Streptococcus lutetiensis* | 3.72E-02 | 1.28E-03 | 5.80E-03 | 1.18 | 5.95E-02 | 1.72E-02 | 2.77E-02 | 1.95E-02 | 4.19E-02 | 8.19E-01 | 2.42E-01 | 1.50E-01 |
| *Chryseobacterium yeoncheonense* | 0 | 0 | 0 | 0 | 2.29E-03 | 0 | 1.80 | 2.47E-01 | 3.49E-03 | 4.21E-01 | 3.95E-02 | 9.86E-03 |
| *Bacteroides fragilis* | 0 | 1.28E-03 | 0 | 2.02E-02 | 0 | 3.00E-02 | 0 | 5.14E-02 | 4.53E-02 | 5.31E-02 | 8.15E-02 | 2.14 |
| *Lactococcus raffinolactis* | 2.36E-02 | 1.66E-02 | 1.06E-01 | 4.05E-02 | 3.69E-01 | 8.58E-03 | 2.34E-01 | 6.47E-01 | 0 | 3.56E-01 | 5.88E-02 | 5.26E-01 |
| unclassified *Vicinamibacteraceae* | 1.12E-02 | 8.96E-03 | 7.52E-01 | 1.82E-01 | 4.58E-03 | 1.72E-01 | 2.00E-02 | 0 | 1.85E-01 | 4.13E-02 | 4.28E-01 | 5.78E-01 |
| *Streptococcus thermophilus* | 0 | 0 | 0 | 3.64E-02 | 1.78 | 0 | 9.25E-03 | 1.15E-01 | 2.44E-02 | 3.02E-01 | 2.35E-02 | 5.07E-02 |
| unclassified *Odoribacter* | 0 | 0 | 0 | 5.26E-02 | 0 | 5.36E-01 | 0 | 2.06E-03 | 7.60E-01 | 2.36E-02 | 7.95E-01 | 1.37E-01 |
| *Enterobacter cloacae* | 0 | 0 | 2.24 | 0 | 0 | 0 | 0 | 0 | 0 | 0 | 0 | 9.39E-04 |
| *Epilithonimonas hominis* | 6.33E-02 | 1.28E-03 | 3.07E-01 | 0 | 4.33E-01 | 1.72E-02 | 5.36E-01 | 2.57E-02 | 3.49E-03 | 3.78E-01 | 2.27E-02 | 4.07E-01 |
| *Bacillus subtilis* | 1.14E-01 | 5.12E-03 | 5.78E-01 | 8.05E-01 | 4.58E-03 | 1.29E-02 | 1.94E-01 | 7.20E-03 | 1.05E-02 | 1.72E-01 | 3.19E-02 | 1.53E-01 |
| species | G-SN* (%) | X-MN* (%) | X-LT* (%) | S-LN* (%) | XJ* (%) | GD* (%) | ZB* (%) | YT* (%) | JN* (%) | WF* (%) | QD* (%) | DY* (%) |
| *Fusobacterium mortiferum* | 2.22E-01 | 2.07E-01 | 4.57E-01 | 2.43E-02 | 0 | 6.00E-02 | 4.56E-01 | 1.75E-02 | 2.44E-02 | 2.55E-01 | 5.38E-02 | 2.61E-01 |
| *Furfurilactobacillus rossiae* | 0 | 0 | 0 | 8.09E-03 | 0 | 6.60E-01 | 0 | 0 | 6.45E-01 | 3.44E-02 | 4.84E-01 | 1.33E-01 |
| *Atopostipes* sp. | 0 | 3.84E-03 | 1.45E-01 | 2.43E-02 | 1.50 | 0 | 1.79E-01 | 1.03E-03 | 0 | 4.82E-02 | 5.88E-03 | 2.91E-02 |
| unclassified *Prevotellaceae* UCG 001 | 0 | 0 | 8.70E-03 | 1.62E-02 | 4.24E-02 | 4.80E-01 | 1.16E-02 | 1.85E-02 | 5.69E-01 | 4.42E-02 | 5.66E-01 | 1.19E-01 |
| unclassified *Alistipes* | 2.61E-02 | 0 | 4.35E-03 | 5.66E-02 | 1.92E-01 | 2.92E-01 | 1.27E-01 | 1.13E-02 | 3.59E-01 | 1.34E-01 | 5.03E-01 | 1.34E-01 |
| *Serratia marcescens* | 9.43E-02 | 6.40E-03 | 3.19E-01 | 4.81E-01 | 4.35E-02 | 1.42E-01 | 1.77E-02 | 2.18E-01 | 1.26E-01 | 1.08E-01 | 1.23E-01 | 1.19E-01 |
| unclassified *Desulfovibrio* | 0 | 0 | 0 | 3.07E-01 | 0 | 1.63E-01 | 0 | 1.85E-02 | 1.43E-01 | 3.54E-02 | 1.02 | 9.81E-02 |
| *Enterococcus faecalis* | 9.93E-03 | 1.28 | 1.45E-03 | 1.50E-01 | 9.27E-02 | 2.57E-02 | 1.54E-02 | 3.91E-02 | 2.09E-02 | 2.16E-02 | 7.48E-02 | 4.55E-02 |
| *Porphyromonas levii* | 0 | 0 | 0 | 2.43E-02 | 8.75E-01 | 0 | 8.38E-01 | 4.11E-03 | 0 | 0 | 0 | 1.41E-03 |
| *Sphingobacterium multivorum* | 5.58E-02 | 6.40E-03 | 0 | 4.05E-03 | 1.03E-02 | 5.06E-01 | 1.25E-01 | 3.09E-03 | 4.50E-01 | 1.12E-01 | 3.34E-01 | 1.20E-01 |
| *Lacticaseibacillus pantheris* | 0 | 0 | 8.70E-03 | 2.83E-02 | 0 | 6.52E-01 | 4.62E-03 | 0 | 5.23E-01 | 1.77E-02 | 3.87E-01 | 1.02E-01 |
| *Staphylococcus delphini* | 1.07 | 1.61E-01 | 5.51E-02 | 1.25E-01 | 6.07E-02 | 0 | 1.31E-01 | 1.03E-03 | 3.49E-03 | 6.00E-02 | 8.41E-04 | 3.90E-02 |
| *Cetobacterium somerae* | 1.07E-01 | 1.45E-01 | 2.64E-01 | 1.21E-02 | 0 | 1.29E-02 | 2.44E-01 | 2.26E-02 | 8.02E-02 | 2.31E-01 | 2.35E-01 | 2.76E-01 |
| *Pasteurella multocida* | 0 | 0 | 0 | 8.09E-03 | 1.54 | 0 | 0 | 0 | 0 | 6.98E-02 | 4.20E-03 | 7.51E-03 |
| *Trueperella pyogenes* | 0 | 0 | 0 | 4.05E-03 | 1.49 | 0 | 4.55E-02 | 0 | 0 | 3.83E-02 | 1.68E-03 | 4.69E-04 |
| *Pantoea dispersa* | 2.73E-02 | 0 | 9.13E-02 | 1.21E-02 | 0 | 3.22E-01 | 1.23E-02 | 5.41E-01 | 2.90E-01 | 1.18E-02 | 1.84E-01 | 6.76E-02 |
| *Rahnella aquatilis* | 7.99E-01 | 0 | 2.90E-03 | 0 | 2.29E-03 | 0 | 3.62E-02 | 2.56E-01 | 0 | 2.46E-01 | 1.01E-02 | 1.99E-01 |
| uncultured *Acidobacteria bacterium* | 1.24E-02 | 8.96E-03 | 4.91E-01 | 2.87E-01 | 6.87E-03 | 5.57E-02 | 2.85E-02 | 0 | 9.42E-02 | 3.24E-02 | 1.68E-01 | 3.55E-01 |
| unclassified [*Eubacterium*] coprostanoligenes group | 9.93E-03 | 0 | 2.46E-02 | 1.58E-01 | 3.18E-01 | 2.06E-01 | 1.64E-01 | 1.11E-01 | 1.19E-01 | 1.53E-01 | 1.53E-01 | 1.17E-01 |
| *Streptococcus hillyeri* | 0 | 2.56E-03 | 1.16E-01 | 1.36 | 0 | 0 | 0 | 0 | 0 | 0 | 0 | 0 |
| *uncultured Bacteroides sp.* | 8.69E-03 | 0 | 8.70E-03 | 0 | 7.56E-01 | 1.72E-02 | 2.69E-01 | 2.06E-03 | 0 | 2.86E-01 | 4.20E-03 | 8.45E-02 |
| *Phocaeicola vulgatus* | 1.24E-03 | 1.15E-02 | 0 | 3.64E-02 | 0 | 3.17E-01 | 0 | 2.06E-02 | 3.52E-01 | 2.74E-01 | 2.60E-01 | 1.37E-01 |
| *Delftia acidovorans* | 3.72E-02 | 1.54E-02 | 1.39E-01 | 4.61E-01 | 8.24E-02 | 3.43E-02 | 3.31E-01 | 5.97E-02 | 3.49E-02 | 1.09E-01 | 2.94E-02 | 5.54E-02 |
| unclassified *Desulfovibrionaceae* | 0 | 0 | 1.45E-03 | 1.66E-01 | 0 | 3.00E-01 | 0 | 6.17E-03 | 1.95E-01 | 7.77E-02 | 3.35E-01 | 2.98E-01 |
| *Companilactobacillus paralimentarius* | 0 | 1.28E-03 | 0 | 7.28E-02 | 0 | 4.42E-01 | 6.94E-03 | 0 | 4.22E-01 | 1.77E-02 | 3.19E-01 | 8.50E-02 |
| species | G-SN* (%) | X-MN* (%) | X-LT* (%) | S-LN* (%) | XJ* (%) | GD* (%) | ZB* (%) | YT* (%) | JN* (%) | WF* (%) | QD* (%) | DY* (%) |
| unclassified *Clostridia* UCG 014 | 6.20E-03 | 2.30E-02 | 2.61E-02 | 1.58E-01 | 1.03E-01 | 1.67E-01 | 6.86E-02 | 1.31E-01 | 1.88E-01 | 1.18E-01 | 3.21E-01 | 5.40E-02 |
| *Faecalibacterium prausnitzii* | 4.96E-03 | 2.56E-03 | 7.25E-03 | 2.22E-01 | 1.37E-02 | 1.33E-01 | 0 | 5.45E-02 | 1.88E-01 | 2.68E-01 | 1.16E-01 | 3.52E-01 |
| *Microbacterium oxydans* | 2.46E-01 | 1.79E-02 | 2.90E-03 | 2.83E-02 | 8.01E-02 | 8.15E-02 | 5.73E-01 | 4.83E-02 | 1.74E-02 | 6.59E-02 | 4.54E-02 | 1.08E-01 |
| unclassified *Christensenellaceae* R 7 group | 8.69E-03 | 1.28E-03 | 3.04E-02 | 4.85E-02 | 3.80E-01 | 4.29E-03 | 3.44E-01 | 0 | 3.14E-02 | 2.72E-01 | 5.72E-02 | 9.76E-02 |
| unclassified TM7a | 0 | 1.28E-03 | 5.80E-03 | 2.83E-02 | 6.87E-02 | 8.15E-02 | 4.12E-01 | 6.17E-03 | 1.22E-01 | 4.22E-01 | 6.47E-02 | 4.65E-02 |
| unclassified *Ulvibacter* | 0 | 2.56E-03 | 0 | 4.45E-02 | 1.72E-02 | 1.14 | 0 | 0 | 1.05E-02 | 0 | 3.36E-02 | 0 |
| *Lachnospiraceae bacterium* DW17 | 0 | 1.28E-03 | 0 | 2.02E-02 | 0 | 3.99E-01 | 0 | 1.95E-02 | 3.63E-01 | 2.56E-02 | 3.29E-01 | 7.70E-02 |
| *Limosilactobacillus reuteri* | 2.73E-02 | 7.30E-02 | 1.15E-01 | 1.17E-01 | 4.58E-03 | 1.37E-01 | 8.17E-02 | 7.30E-02 | 1.88E-01 | 8.75E-02 | 2.77E-01 | 4.79E-02 |
| *Hypsibius dujardini* | 1.24E-03 | 3.84E-03 | 5.65E-02 | 3.24E-02 | 0 | 2.62E-01 | 5.40E-03 | 0 | 4.15E-01 | 2.85E-02 | 3.40E-01 | 8.17E-02 |
| unclassified *Xanthobacteraceae* | 4.84E-02 | 6.40E-03 | 3.91E-02 | 8.09E-01 | 0 | 3.43E-02 | 2.85E-02 | 4.11E-03 | 2.09E-02 | 1.28E-01 | 5.38E-02 | 4.36E-02 |
| *Enterococcus faecium* | 3.72E-03 | 1.28E-03 | 1.16E-01 | 2.83E-02 | 7.10E-02 | 5.15E-02 | 4.43E-01 | 4.22E-02 | 2.79E-02 | 5.21E-02 | 7.31E-02 | 2.97E-01 |
| unclassified UCG 010 | 2.23E-02 | 0 | 2.61E-02 | 5.66E-02 | 3.45E-01 | 3.86E-02 | 3.01E-01 | 1.34E-02 | 2.79E-02 | 1.92E-01 | 5.72E-02 | 1.24E-01 |
| Others | 6.20E-03 | 2.30E-02 | 2.61E-02 | 1.58E-01 | 1.03E-01 | 1.67E-01 | 6.86E-02 | 1.31E-01 | 1.88E-01 | 1.18E-01 | 3.21E-01 | 5.40E-02 |
| Unknown | 4.96E-03 | 2.56E-03 | 7.25E-03 | 2.22E-01 | 1.37E-02 | 1.33E-01 | 0 | 5.45E-02 | 1.88E-01 | 2.68E-01 | 1.16E-01 | 3.52E-01 |

Note: G-SN, buffalo milk from Guangxi; X-MN, horse milk from Xingjiang; X-LT, camel milk from Xinjiang; S-LN, donkey milk from Shandong; XJ, Holstein cow milk from Xinjiang; GD, Holstein cow milk from GuangDong; ZB, Holstein cow milk from Zibo; YT, Holstein cow milk from Yantai; JN, Holstein cow milk from Jinan; WF, Holstein cow milk from Weifang; QD, Holstein cow milk from QingDao; DY, Holstein cow milk from Dongying.
