# Supplementary material for: Mapping bacterial microbiota variations in raw milk: geographic and type-specific insights
Source: Microbiol Spectr. 2025 Oct 27;13(12):e00933-25. doi: 10.1128/spectrum.00933-25 (PMC12671074; doi:10.1128/spectrum.00933-25)
Supplement: Table S6 — Relative abundance of lactic acid bacteria at the species level of raw milk from different regions and types (>0.1%). [file spectrum.00933-25-s0007.docx]

| species | G-SN (%) | X-MN (%) | X-LT (%) | S-LN (%) | XJ (%) | GD (%) | ZB (%) | YT (%) | JN (%) | WF (%) | QD (%) | DY (%) |
| --- | --- | --- | --- | --- | --- | --- | --- | --- | --- | --- | --- | --- |
| *Lactobacillus helveticus* | 4.38E-01 | 4.14E-01 | 2.89 | 5.55 | 7.68E-01 | 5.15E-02 | 1.98 | 2.85E-01 | 1.26E-01 | 1.49 | 1.14E-01 | 7.92E-03 |
| *Limosilactobacillus pontis* | 3.28E-01 | 1.15E-02 | 3.25 | 1.08 | 0 | 9.01E-02 | 1.69 | 2.37E-02 | 2.09E-02 | 7.21E-01 | 5.30E-02 | 1.09E-02 |
| *Lactococcus lactis* | 2.06E-01 | 6.79E-02 | 1.35E-01 | 2.51E-01 | 1.03 | 4.42E-01 | 3.54E-01 | 1.84E-01 | 3.98E-01 | 4.69E-01 | 3.61E-01 | 1.36E+00 |
| *Lactobacillus acetotolerans* | 1.64E-01 | 7.68E-03 | 1.61 | 1.54 | 2.29E-03 | 8.58E-03 | 9.47E-01 | 9.36E-02 | 0 | 6.82E-01 | 5.88E-03 | 4.30E-01 |
| *Bacillus subtilis* | 1.14E-01 | 5.12E-03 | 5.78E-01 | 8.05E-01 | 4.58E-03 | 1.29E-02 | 1.94E-01 | 7.20E-03 | 1.05E-02 | 1.72E-01 | 3.19E-02 | 1.50E-01 |
| *Lentilactobacillus hilgardii* | 1.13E-01 | 1.15E-02 | 7.74E-01 | 1.85 | 0 | 0 | 5.20E-01 | 4.01E-02 | 0 | 3.69E-01 | 8.41E-04 | 1.70E-01 |
| *Ligilactobacillus murinus* | 4.72E-02 | 9.35E-02 | 1.10E-01 | 3.80E-01 | 1.14E-03 | 2.70E-01 | 1.13E-01 | 1.85E-02 | 3.98E-01 | 9.63E-02 | 2.00 | 1.60E-01 |
| *Lentilactobacillus buchneri* | 5.34E-02 | 1.15E-02 | 3.91E-01 | 2.51 | 6.87E-03 | 1.59E-01 | 2.38E-01 | 8.95E-02 | 3.59E-01 | 1.90E-01 | 1.91E-01 | 1.40E-01 |
| *Limosilactobacillus reuteri* | 2.73E-02 | 7.30E-02 | 1.15E-01 | 1.17E-01 | 4.58E-03 | 1.37E-01 | 8.17E-02 | 7.30E-02 | 1.88E-01 | 8.75E-02 | 2.77E-01 | 5.00E-02 |
| *Levilactobacillus brevis* | 2.36E-02 | 5.12E-03 | 1.25E-01 | 2.25 | 0 | 1.35 | 7.86E-02 | 1.03E-02 | 1.34 | 6.59E-02 | 1.07 | 3.70E-01 |
| *Lactococcus raffinolactis* | 2.36E-02 | 1.66E-02 | 1.06E-01 | 4.05E-02 | 3.69E-01 | 8.58E-03 | 2.34E-01 | 6.47E-01 | 0 | 3.56E-01 | 5.88E-02 | 5.30E-01 |
| *Enterococcus faecalis* | 9.93E-03 | 1.28 | 1.45E-03 | 1.50E-01 | 9.27E-02 | 2.57E-02 | 1.54E-02 | 3.91E-02 | 2.09E-02 | 2.16E-02 | 7.48E-02 | 4.55E-04 |
| *Leuconostoc mesenteroides* | 1.36E-02 | 6.27E-02 | 9.30 | 4.45E-02 | 1.27E-01 | 5.53E-01 | 1.77E-02 | 5.04E-02 | 4.22E-01 | 8.58E-01 | 3.69E-01 | 2.00E-01 |
| *Enterococcus faecium* | 3.72E-03 | 1.28E-03 | 1.16E-01 | 2.83E-02 | 7.10E-02 | 5.15E-02 | 4.43E-01 | 4.22E-02 | 2.79E-02 | 5.21E-02 | 7.31E-02 | 3.00E-01 |
| *Lactiplantibacillus plantarum* | 4.96E-03 | 1.28E-03 | 1.01E-02 | 4.13E-01 | 3.43E-03 | 2.98E+01 | 1.00E-02 | 2.37E-02 | 3.89E+01 | 7.18E-01 | 2.27E+01 | 1.08E+01 |
| *Lactobacillus vaccinostercus* DSM 20634 | 3.72E-03 | 0 | 5.80E-03 | 6.47E-02 | 1.14E-03 | 1.13 | 5.40E-03 | 3.09E-03 | 1.18 | 6.78E-02 | 1.05 | 3.30E-01 |
| *Lactobacillus johnsonii* | 4.96E-03 | 2.56E-03 | 7.25E-03 | 2.87E-01 | 1.26E-02 | 1.29E-01 | 1.54E-02 | 2.47E-02 | 9.07E-02 | 1.32E-01 | 6.26E-01 | 1.71 |
| *Furfurilactobacillus rossiae* | 0 | 0 | 0 | 8.09E-03 | 0 | 6.60E-01 | 0 | 0 | 6.45E-01 | 3.44E-02 | 4.84E-01 | 1.30E-01 |
| *Companilactobacillus paralimentarius* | 0 | 0 | 0 | 8.09E-03 | 0 | 6.60E-01 | 0 | 0 | 6.45E-01 | 3.44E-02 | 4.84E-01 | 8.00E-02 |
| *Lactococcus piscium* | 0 | 0 | 0 | 0 | 3.38E-01 | 1.29E-02 | 2.47E-02 | 2.68 | 0 | 2.28 | 2.41E-01 | 7.10E-01 |
| *Carnobacterium maltaromaticum* | 4.96E-03 | 0 | 2.90E-03 | 0 | 1.03E-02 | 1.72E-02 | 7.56 | 3.99E-01 | 0 | 3.15E-02 | 5.88E-03 | 5.00E-02 |
| *Streptococcus thermophilus* | 0 | 0 | 0 | 3.64E-02 | 1.78 | 0 | 9.25E-03 | 1.15E-01 | 2.44E-02 | 3.02E-01 | 2.35E-02 | 5.00E-02 |

Table S6 Relative abundance of lactic acid bacteria at the species level of raw milk from different regions and types (＞0.1%).

Note: G-SN, buffalo milk from Guangxi; X-MN, horse milk from Xingjiang; X-LT, camel milk from Xinjiang; S-LN, donkey milk from Shandong; XJ, Holstein cow milk from Xinjiang; GD, Holstein cow milk from Guangdong; ZB, Holstein cow milk from Zibo; YT, Holstein cow milk from Yantai; JN, Holstein cow milk from Jinan; WF, Holstein cow milk from Weifang; QD, Holstein cow milk from Qingdao; DY, Holstein cow milk from Dongying.
